# Ultrafast Interfacial Charge Transfer Initiates Mechanical Stress and Heat Transport at the Au‐TiO_2_ Interface

**DOI:** 10.1002/advs.202400919

**Published:** 2024-07-08

**Authors:** Jun Heo, Alekos Segalina, Doyeong Kim, Doo‐Sik Ahn, Key Young Oang, Sungjun Park, Hyungjun Kim, Hyotcherl Ihee

**Affiliations:** ^1^ Center for Advanced Reaction Dynamics (CARD) Institute for Basic Science (IBS) Daejeon 34141 Republic of Korea; ^2^ Radiation Center for Ultrafast Science Korea Atomic Energy Research Institute (KAERI) Daejeon 34057 Republic of Korea; ^3^ Department of Chemistry Korea Advanced Institute of Science and Technology (KAIST) Daejeon 34141 Republic of Korea; ^4^ Present address: Samsung Electronics Republic of Korea

**Keywords:** hot carrier dynamics, interfacial charge transfer, interfacial coupling, metal‐semiconductor interface, ultrafast electron diffraction, ultrafast heat transfer

## Abstract

Metal‐semiconductor interfaces are crucial components of optoelectronic and electrical devices, the performance of which hinges on intricate dynamics involving charge transport and mechanical interaction at the interface. Nevertheless, structural changes upon photoexcitation and subsequent carrier transportation at the interface, which crucially impact hot carrier stability and lifetime, remain elusive. To address this long‐standing problem, they investigated the electron dynamics and resulting structural changes at the Au/TiO_2_ interface using ultrafast electron diffraction (UED). The analysis of the UED data reveals that interlayer electron transfer from metal to semiconductor generates a strong coupling between the two layers, offering a new way for ultrafast heat transfer through the interface and leading to a coherent structural vibration that plays a critical role in propagating mechanical stress. These findings provide insights into the relationship between electron transfer and interfacial mechanical and thermal properties.

## Graphical abstract

Their research investigates the dynamics of metal‐semiconductor interfaces, crucial for optoelectronic and electrical devices. Using UED, they reveal how interlayer electron transfer at the Au/TiO_2_ interface induces strong coupling, enabling ultrafast heat transfer and coherent structural vibrations. These insights elucidate the interplay between electron dynamics and mechanical/thermal properties at interfaces and deepens understanding of relaxation mechanisms in photogenerated hot charge carriers, with potential impact on energy conversion and catalysis.

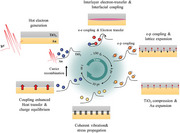

## 1 Introduction

Hot electrons, also referred to as energetic electrons, play pivotal roles for the functionality and efficiency of optoelectronic and electrical devices.^[^ ^1^, ^2^, ^3^ ^]^ These electrons gain extra energy beyond the Fermi level from external disturbances. Their presence within these devices has a profound impact on various processes, encompassing charge transport, energy dissipation, and chemical reactions.^[^ ^4^, ^5^ ^]^ While hot electrons offer advantages for efficient energy harvesting and catalytic reactions, they also introduce drawbacks that can influence device performance.^[^ ^6^, ^7^, ^8^ ^]^ In this context, exploring the relaxation dynamics of hot electrons from a structural perspective becomes imperative, and gaining an understanding of how hot electron relaxation influences the structural integrity and properties of the device materials is crucial for optimizing device design and functionality.^[^ ^9^ ^]^ In optoelectronic and photovoltaic applications,^[^ ^10^, ^11^, ^12^, ^13^, ^14^, ^15^ ^]^ the transfer and relaxation efficiencies of carriers at the material interface, where the heterojunction exists, are crucial determinants of performance.^[^ ^16^, ^17^ ^]^ Therefore, the dynamics at the heterojunction as well as those of the bulk materials comprising the material interface need to be understood, but the mechanisms and effects of interfacial hot carrier transfer are not yet fully comprehended.

Metal‐semiconductor heterojunctions stand out among heterojunction systems, particularly due to their relevance in hot electron phenomena.^[^ ^18^, ^19^ ^]^ Heterojunction systems are also known as prototypical systems of the hot electron transfer phenomenon. In this system, the formation of a Schottky barrier at the interface generates an energy gradient, enabling hot electrons from the metal layer to transfer spontaneously to the semiconductor layer. Metal‐semiconductor heterojunctions, with their inherent driving force for hot electron transfer, provide a promising platform for the development of efficient photovoltaic cells, including hot carrier photovoltaics. These heterojunctions facilitate the effective collection and injection of hot electrons across the interface, making them a valuable asset in the advancement of photovoltaic technology.

Numerous studies have delved into carrier transport and structural alterations at metal‐semiconductor heterojunction interfaces using diverse techniques, such as optical,^[^ ^20^, ^21^ ^]^ UV,^[^ ^22^ ^]^ XUV,^[^ ^23^ ^]^ X‐ray spectroscopies,^[^ ^24^ ^]^ as well as diffraction.^[^ ^24^, ^25^ ^]^ However, the intricate interface structure poses challenges in observing charge transfer dynamics and structural changes. While spectroscopic methods have successfully addressed electronic dynamics at the metal‐semiconductor interface, they are sensitive to the energy of the electrons within the components at the interface, making it difficult to separate signals from the metal and semiconductor layers. Recent studies have shown that X‐ray absorption spectroscopy can reveal electronic and structural dynamics at the Fe‐MgO interface, and the dynamics at the Fe‐MgO interface are driven by electron–electron (e–e) scattering rather than electron transfer.^[^ ^24^ ^]^ In another study, although diffraction studies were carried out, they were predominantly focused on metal‐metal interfaces such as the Au‐Ni interface.^[^ ^25^ ^]^ As evident from these examples, investigations into the dynamics of interfacial charge transfer are rare, especially concerning structural aspects. This was due to the intricate interplay between electron and lattice dynamics and the challenge of simultaneously analyzing two distinct layers.^[^ ^24^, ^26^ ^]^

To address this difficulty, we chose the Au/TiO_2_ heterojunction system because the diffraction peaks from Au and TiO_2_ are well separated. In fact, this prototypical metal‐semiconductor heterojunction system has been extensively studied for its photophysical and photochemical properties, and its structural variants have been interrogated in the context of structure‐function relations.^[^ ^18^, ^27^, ^28^ ^]^ Furthermore, when plasmonic gold nanostructures are combined with TiO_2_, the photocatalytic activities and photoconversion efficiencies improve significantly.^[^ ^29^ ^]^ Despite the extensive studies on the Au/TiO_2_ junction system, however, the detailed energy flow and associated structural changes underlying the enhanced photoconversion efficiency remain unclear. In this work, we used UED^[^ ^26^, ^30^, ^31^, ^32^, ^33^, ^34^, ^35^, ^36^, ^37^ ^]^ to investigate the role of photo‐induced charge carriers in the heterojunction Au/TiO_2_ photocatalyst and monitored the subsequent deformation of lattice structures and coherent lattice motions. The UED data unveiled the mechanical motion and electrical property changes accompanied by the interlayer charge transfer between the metal and semiconductor.

## 2 Results and Discussion

### 2.1 Sample Characterization

The Au and TiO_2_ layers were characterized using UV‐visible absorbance measurements, XRD (X‐ray diffraction), cross‐sectional SEM (scanning electron microscopy), TEM (transmission electron microscopy), and EDS (elemental energy‐dispersive X‐ray spectroscopy) (Figure S1). The UV‐visible spectrum exhibits an absorption band around 800 nm, attributed to the plasmonic structure. Comparing the UV‐visible spectra of plasmonic Au (15‐nm Au) and Au film (30‐nm Au) reveals that plasmonic Au exhibits stronger absorbance than the Au film (Figure S1a). A similar increase in absorbance at 800 nm due to plasmonic Au is also observed in the Au/TiO_2_ sample, where a TiO_2_ layer is placed on plasmonic Au, indicating that the 800 nm absorption in the Au/TiO_2_ sample is associated with plasmonic absorption characteristics.^[^ ^38^, ^39^ ^]^ The TEM image of the Au/TiO_2_ sample (Figure S1b) confirms the presence of a dispersed plasmonic structure within the Au domain. EDS mapping images of Au, Ti, and O for the Au/TiO_2_ sample demonstrate the uniform formation of the TiO_2_ layer and the plasmonic structure of the Au layer (Figure S1c). To analyze the thicknesses of the Au and TiO_2_ layers, a cross‐sectional SEM image was obtained (Figure S2). The image shows that the 15‐nm Au film possesses a plasmonic structure with a width of tens of nm and a thickness of 15 nm, while the TiO_2_ layer has a thickness of 20 nm.

### 2.2 UED Experiment

**Figure 1:**
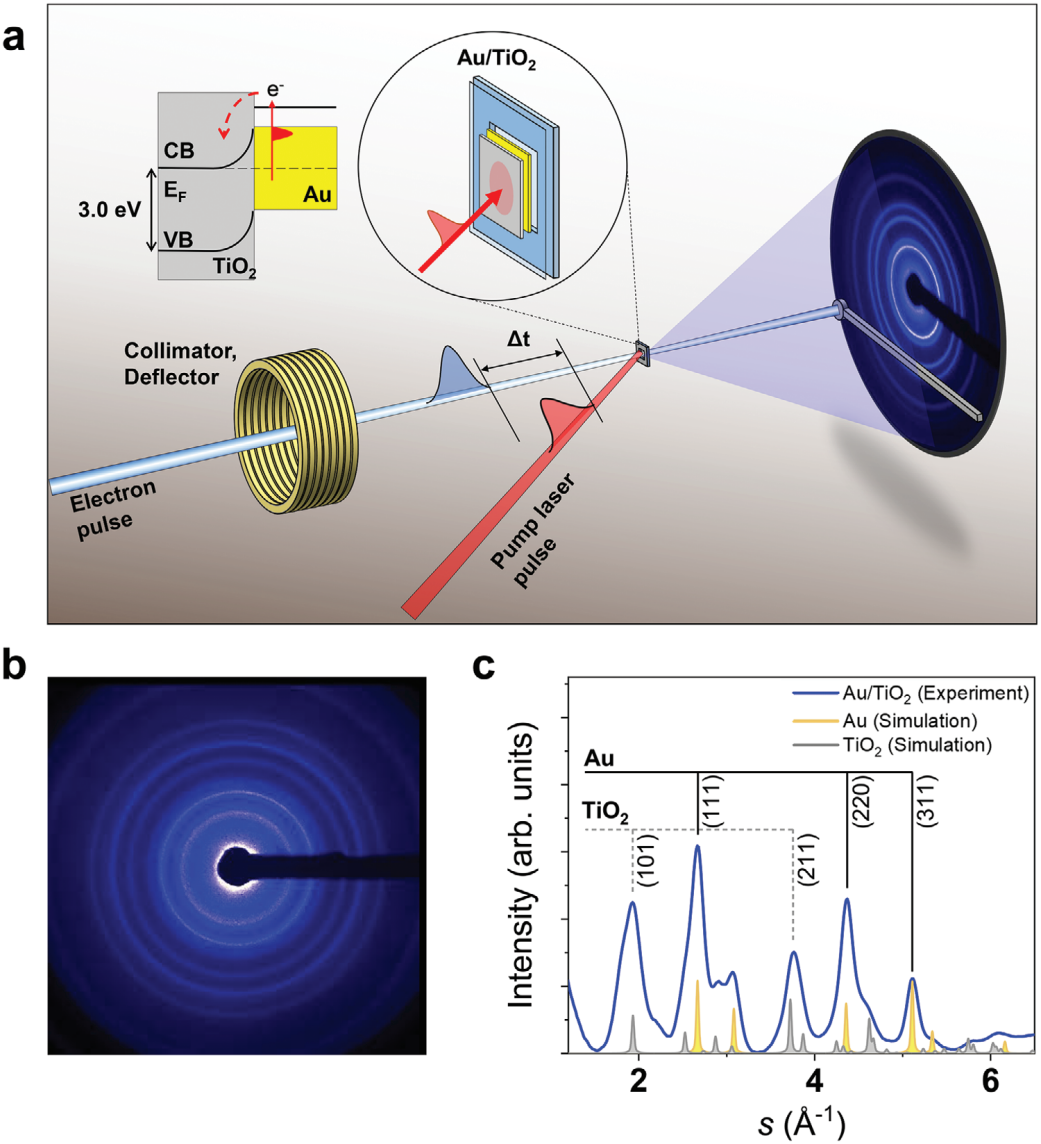
Diagram and diffraction data from UED setup. (a) Schematic of the UED experiment used to capture charge transfer induced structural motion in a metal‐semiconductor heterojunction system. The electron pulse was accelerated to 90 keV and compressed to ∼400 fs. The resulting electron pulse was directed to the sample of ∼40 nm thickness. The diffraction pattern of the scattered electrons was collected by an ICCD camera coupled with a phosphor screen which was placed ∼40 cm downstream from the sample. A pump‐laser beam at 800 nm excited the electrons in the Au domain, generating hot electrons. The schematic diagram on the top left illustrates the interlayer electron transfer from Au to TiO_2_. Since the bandgap in the TiO_2_ is higher than the energy of the photons in the pump laser pulse, hot electrons cannot be generated in TiO_2_ upon pump irradiation. The photo responses were interrogated by a time‐delayed electron pulse. (b) A diffraction image of the sample is shown. The dark region in the center that extends towards the right‐hand side of the image is the shade of the beam block. (c) 1D diffraction curve at ‐100 ps from the Au/TiO_2_ (black) and comparison with calculated powder diffraction peaks (yellow for Au and gray for TiO_2_). The Miller indices for the observed peaks are indicated.

**Figure 2:**
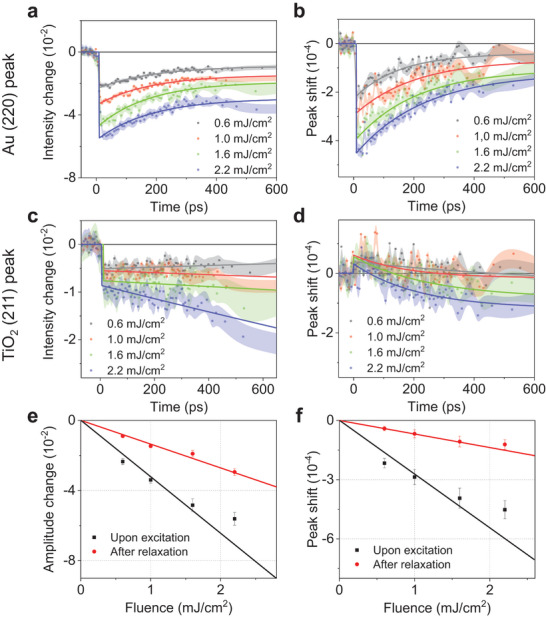
Power‐dependent dynamics of the Au/TiO_2_ junction system and corresponding peak intensity changes and peak shifts upon photoexcitation. (a, c) Peak intensity changes of (a) Au (220) and (c) TiO_2_ (211) with the standard deviations as error bars (shaded region) are presented. (b, d) Peak shifts of (b) Au (220) and (d) TiO_2_ (211) with the standard deviations as error bars (shaded region) are presented. **(e)** Power dependence on the intensity of the Au (220) peak upon excitation (black, before 10 ps) and after relaxation (red, at equilibrium, after 400 ps). The error bars indicate the standard deviations. **(f)** Power dependence on the peak shifts of the Au (220) peak upon excitation (black) and after relaxation (red), with the standard deviations as error bars.

The UED experiment was conducted using a home‐built setup. The structural changes of the bilayer system upon irradiation at 800 nm (∼1.54 eV) were tracked over a wide time range from −15 ps to 500 ps using an electron pulse accelerated to 90 KeV (**Figure** **1**a). A pump laser pulse at 800 nm was used to excite the sample, resulting in the generation of hot electrons in the Au layer through localized surface plasmon resonance (LSPR) (Figure 1a).^[^ ^40^, ^41^, ^42^, ^43^, ^44^, ^45^ ^]^ In contrast, rutile TiO_2_, with its larger bandgap (∼3.0 eV), does not exhibit absorption at 800 nm, resulting in the absence of hot electron generation in the TiO_2_ layer (Figure 1a). Consequently, no significant difference signal was observed in the time‐resolved UED data upon 800‐nm laser irradiation on the TiO_2_‐only sample (Figure S3). A typical 2D electron diffraction image of Au/TiO_2_ displaying a Debye‐Scherrer ring pattern is shown in Figure 1b. The 2D diffraction images obtained at different time delays were azimuthally integrated to generate 1D diffraction patterns. The inelastic scattering signal was then removed from each 1D diffraction pattern using the wavelet transform method.^[^ ^46^ ^]^ The resulting diffraction curve is shown in **Figure** **2**b. To ensure that the used pump fluence exclusively corresponds to the one‐photon process, without any multiphoton excitation or other nonlinear effects such as the generation of hot electrons in TiO_2_, the time‐resolved data were scanned at multiple laser fluences ranging from 0.6 to 2.2 mJ/cm^2^. This range was carefully chosen to include the region of linear photoabsorption.^[^ ^31^ ^]^

### 2.3 Thermal and Structural Motions

The diffraction peaks of the Au layer are well separated from those of the TiO_2_ layer, allowing for the extraction of the structural dynamics of each layer by tracking the separated diffraction peaks (Figure 1c). By comparing the experimentally obtained diffraction curve with the theoretically calculated Au and TiO_2_ diffraction peaks,^[^ ^47^ ^]^ the (111), (200), (220), and (311) peaks of Au, as well as the (101) and (211) peaks of TiO_2_, were identified. These peaks are distinct and exhibit strong intensities, making them suitable for tracking their peak intensities and peak positions. The experimental diffraction peaks were fitted using Gaussian functions to obtain the intensities and positions of the peaks.^[^ ^48^, ^49^, ^50^, ^51^, ^52^ ^]^ To investigate the time‐dependent changes in the diffraction data, the intensity changes (Δ*I*(*t*)*/I*_0_) and peak shifts (Δ*S*(*t*)*/S*_0_) of each peak were quantified. Here, Δ*I*(*t*) and Δ*S*(*t*) represent the changes in peak intensity and position, respectively, at a specific time delay, *t*, following photoexcitation. *I*_0_ and *S*_0_ correspond to the peak intensity and position, respectively, at the negative reference time delay of ‐30 ps, serving as a baseline reference for analyzing and comparing subsequent changes in peak characteristics at different time delays.

Figures 2a,b depict the time‐resolved intensity changes and peak shifts of the Au (220) peak at various fluences, while Figure 2c,d illustrate those of the TiO_2_ (211) peak. The plots demonstrate that the intensities of both the Au (220) and TiO_2_ (211) peaks exhibit rapid drops immediately after time zero, indicating an increase in material temperature. Upon excitation, the hot electrons generated in Au undergo relaxation through electron–electron (e–e) and electron–phonon (e–p) couplings, which influence the structure and temperature of the Au layer. The temperature change caused by the hot electrons affects the position and intensity of the Au diffraction peaks. For example, the loss of periodicity due to increased temperature and thermal motion leads to a decrease in peak intensity as it reflects the structure's periodicity.^[^ ^31^, ^52^, ^53^ ^]^ Thus, the temperature changes of the sample can be calculated from the intensity changes of the diffraction signal (Δ*I*(*t*)*/I*_0_ ∝ Δ*T*). On the other hand, Δ*S*(*t*)*/S*_0_ directly indicates the changes in the lattice structure of the domains. According to the Laue condition, the amplitude of the reciprocal lattice vector, which determines the position of the diffraction peak, is inversely proportional to the lattice spacing. Therefore, a peak shift in the negative direction indicates lattice expansion, while a shift in the positive direction indicates lattice contraction.

Prior to the analysis of structural and thermal dynamics in both the Au and TiO_2_ domains, we initially explored the dependence of the peak intensities of Au—where hot electrons are generated—on laser fluence to identify linear photoabsorption. For this, the Δ*I*(*t*)*/I*_0_ and Δ*S*(*t*)*/S*_0_ of the Au (220) peak were plotted as a function of laser fluence (Figure 2e,f). Since the phonon–phonon (p–p) coupling occurs in the time scale of 10 to 100 ps,^[^ ^54^ ^]^ the abrupt changes in Δ*I*(*t*)*/I*_0_ and Δ*S*(*t*)*/S*_0_ in the early time region (0 ∼ 10 ps), immediately after excitation, are mainly influenced by the e–p coupling within the Au domain. Hence, the temperature and lattice structure changes observed in this time region directly reflect the number of generated hot electrons. On the contrary, after 10 ps, as the p–p coupling takes effect, the dynamics of the Au domain are also influenced by the TiO_2_ domain due to their physical contact. To comprehensively analyze the influence of hot electrons, a comparison was made between the data from two time regions: upon excitation (before 10 ps) and after relaxation (after 400 ps). After relaxation, both Δ*I*(*t*)*/I*_0_ and Δ*S*(*t*)*/S*_0_ exhibit a linear response to fluence (Figure 2e,f), indicating that the total amount of energy absorbed by Au exhibits a linear response to changes in pump laser fluence. In contrast, those changes observed in the “upon excitation” region exhibit a non‐linear dependency, primarily attributed to the transient structural motion and interfacial coupling between the Au and TiO_2_ layers. This phenomenon will be further explored in the subsequent sections.

To quantitatively analyze the time‐dependent changes in Δ*I*(*t*)*/I*_0_ and Δ*S*(*t*)*/S*_0_ of the Au (220) peak, a fitting procedure was applied to each time profile. In the fitting model, the kinetics were described using an exponential function, while the instrument response function and the position of time zero were accounted for using a Heaviside step function. The fitted parameters, exponential rise time constant of Au peak shift and their corresponding intensity changes are listed in Table S1.

### 2.4 Lattice expansion and contraction

Upon excitation, both Au (220) and TiO_2_ (211) peaks exhibit rapid drops in peak intensities within 4 ps, indicating a corresponding increase in the temperature (Figure 2a,c). Since TiO_2_ does not absorb 800‐nm photons, any temperature change observed in TiO_2_ must originate from energy transferred from Au. There are two possible pathways for the energy transfer: (i) hot electrons, transferred from Au to TiO_2_, are coupled to the TiO_2_ lattice via e–p coupling, or (ii) heat is transferred from Au to TiO_2_ through the p–p coupling. Considering that p–p coupling generally occurs on a slower time scale (10 to 100 ps)^[^ ^54^ ^]^ compared to the observed rapid temperature change, the notable temperature rise of TiO_2_ occurring within merely 4 ps after excitation can be attributed solely to the e–p coupling. The hot electrons responsible for this e–p coupling in TiO_2_ should be transferred from Au. This assignment is consistent with a previous study that reported the transfer of hot electrons from Au to the TiO_2_ layer within 100 fs.^[^ ^55^ ^]^

As depicted in Figure 2a,c, Au and TiO_2_ exhibit opposite behaviors after the initial rapid drops in peak intensity. While Au shows a recovery in peak intensity, TiO_2_ has a continued decrease. Since the Δ*I*(*t*)*/I*_0_ of peaks represents the temperature change of the corresponding domain, the observed recovery and the continued decrease of peak intensity in Au (220) and TiO_2_ (211), respectively, suggest that whereas Au undergoes a thermal relaxation process to reach thermal equilibrium, the TiO_2_ domain continues to heat up. The continued heating in TiO_2_ suggests that the heat generated in the Au layer is being transferred to the TiO_2_ layer. It is noteworthy that the heating of TiO_2_ occurs at a slower rate compared to the thermal relaxation of Au, as shown in Figure 2a,c, and Table S1. The delayed rise in the temperature of TiO_2_ implies that the low thermal conductivity of TiO_2_ impedes its ability to quickly reach thermal equilibrium within the TiO_2_ domain itself.^[^ ^56^ ^]^ These heat transfer and temperature changes in this system will be discussed in the “Interfacial coupling” section by comparing the dynamics of Au within Au/TiO_2_ and Au‐only samples.

The evolution of Δ*S*(*t*)*/S*_0_, illustrating alterations in the unit cell's structure, reveals unique dynamics that contrast with the behavior observed in Δ*I*(*t*)*/I*_0_. While the dynamics in Δ*I*(*t*)*/I*_0_ demonstrate an increase in temperature, which accompanies lattice expansion, Δ*S*(*t*)*/S*_0_ reflects intricate variations in structural configuration over time. In Figure 2b,c, TiO_2_ (211) and Au (220) peaks shift in opposite directions. Following excitation, the Au (220) peak undergoes an initial shift towards the low *s* region and subsequently returns to the high *s* region during relaxation. In contrast, the TiO_2_ (211) peak initially shifts towards the high *s* region and gradually returns to the low *s* region over time. Given that peak shifts towards the high (Δ*S*(*t*)*/S*_0_ > 0) and low (Δ*S*(*t*)*/S*_0_ < 0) *s* regions correspond to lattice contraction and expansion, respectively, the shift in the TiO_2_ peak suggests that the TiO_2_ lattice contracts after photoexcitation and gradually expands over time.

### 2.5 Coherent Motion

To enhance our comprehension of the lattice contraction's origin in TiO_2_, we conducted further investigations by collecting additional UED data with finer time steps within the time range of ‐15 ps to 70 ps. The pump fluence used was 1.6 mJ/cm^2^, chosen deliberately as it fell within the region where linear power dependence was observed.

**Figure 3:**
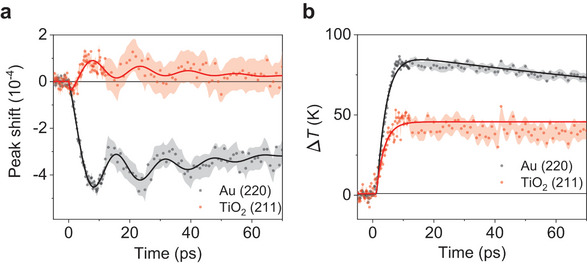
Peak shifts and temperature changes in Au/TiO_2_ sample upon photoexcitation (a, b) Time‐resolved peak shifts (a) and temperature changes (b) of Au (220) (black dot) and TiO_2_ (211) (red dot) peaks of Au/TiO_2_ sample upon photoexcitation (1.6 mJ/cm^2^). The fitted results are shown as solid lines, with standard deviations indicated by the shaded areas.

**Figure** **3**a presents the Δ*S*(*t*)*/S*_0_ curves for the Au (220) and TiO_2_ (211) peaks, and Figure 3b illustrates their corresponding Δ*T*(*t*), which were calculated from Δ*I*(*t*)*/I*_0_. It should be highlighted that the Δ*S*(*t*)*/S*_0_ of the TiO_2_ (211) peak exhibits features not observed in the rough‐scan data with coarser time delays: (i) a noticeable shift to the negative region (lower *s* region) immediately after time zero, and (ii) a subsequent, oscillatory pattern, indicative of coherent vibration. Intriguingly, a similar but anticorrelated oscillation is observed in the Δ*S*(*t*)*/S*_0_ of the Au (220) peak, indicating that the vibrational motion within the Au layer is found to be anticorrelated with that of the TiO_2_ layer. On the other hand, the Δ*T*(*t*) of both Au (220) and TiO_2_ (211) immediately increases after time zero, suggesting that both layers experience instantaneous temperature changes due to the e–p coupling.

The structural evolution of TiO_2_ unfolds through a series of distinct steps. Initially, within a span of 4 ps a pronounced expansion takes place, marked by a discernible negative peak shift as depicted in Figure 3a. This expansion is triggered by the injection of hot electrons, which instigate an expansion of TiO_2_ cells and consequently induce a negative shift in the TiO_2_ (211) peak. Concurrently, these transferred electrons make TiO_2_ more deformable and increasingly susceptible to compressive forces, as evidenced in Table S3. Subsequently, as the thermal expansion of Au proceeds and the interfacial p–p coupling strengthens (see “Interfacial coupling” and “Computational analysis” sections for details), the expanding domain of Au begins to exert a constraining influence on the further expansion of TiO_2_. This results in a step of contraction, evidenced by the positive peak shift observed in Figure 2d, where the TiO_2_ lattice undergoes compression. Thus, the suppressing effect exerted by the expanding Au domain on TiO_2_ induces contraction and the stress built at the interface starts the coherent vibration. Following this stress‐induced lattice contraction and coherent vibration, depicted in Figure 3a, the mechanical stresses that had physically constrained the TiO_2_ lattice are gradually relieved at the interface. Consequently, the thermal energy stored within Au is slowly transferred to TiO_2_, initiating a gradual lattice expansion accompanied by a negative peak shift. This slow expansion signifies the restoration of equilibrium within the system.

In order to quantitatively characterize the structural dynamics, we conducted fitting analyses on the temporal behavior of key features, including peak shift and temperature changes, within the Au (220) and TiO_2_ (211) peaks. First, for the fitting of the peak shifts, Δ*S*(*t*)*/S*_0_, the sums of exponentials and damping sine function shared for both peaks were used. Fitted results are shown in Figure 3a and Table S2. The assumptions and parameters used for the fitting process are as follows: i) The expansion and vibrational motion of Au induce the contraction and vibrational motion of TiO_2_. As a result, the mechanical motion of Au and TiO_2_ is closely anti‐correlated to each other after 4 ps, sharing the rising time constant (t_S2_), vibrational period (T_S_), and damping constant of vibration (t_d_). ii) Alterations in Au do not impact the time constant for TiO_2_’s initial expansion (t_S1_). iii) Each domain relaxes with an independent decay constant, which is determined from the rough scan data (Figure 2a,c), as shown in Table S1 (t_S3_). As evident in Figure 3a, the fitted curves satisfactorily describe the experimental data, effectively characterizing the system's behavior. The temperature changes, Δ*T*(*t*), show the monotonous dynamics without any oscillatory features: while Au (220) displays both decay (t_A1_) and rise (t_A2_) profiles, TiO_2_ (211) only demonstrates a monotonic decay (t_A1_). Here, we used only a single exponential for Δ*T*(*t*) of TiO_2_ because the further decay of Δ*T*(*t*) of TiO_2_ (211) occurs with a much longer time constant (Figure 2c), which falls outside the time range depicted in Figure 3b. For Au (220), due to the incomplete depiction of the rising profile in the available data (Figure 3b), we incorporated the fitted rise constant of Δ*T*(*t*) of Au (220) derived from the coarse scan data to describe the rising profile (Table S1). The fitted results on the Δ*T*(*t*) of each peak are shown in Figure 3b, with the corresponding parameters detailed in Table S2. For the intensity change of Au, the time constant of the initial temperature change (t_A1,Au_ = 3.05 ± 0.13 ps) of Au (220) agrees with the reported values from the UED studies on pure Au films (3 ∼ 4 ps).^[^ ^31^, ^50^ ^]^ The observed rise time constant of TiO_2_ (t_A1,TiO2_ = 2.62 ± 0.35 ps) is faster than that of Au (t_A1,Au_ = 3.05 ± 0.13 ps).

Following the initial transient lattice expansion (t_S1_ = 0.41 ± 0.34 ps), TiO_2_ undergoes a lattice contraction and coherent vibration reflected by Δ*S*(*t*)*/S*_0_. In contrast, the Au (220) peak undergoes a monotonous lattice expansion before exhibiting vibrational motion that is anticorrelated with that of TiO_2_. Generally, the temperature change, Δ*T*(*t*), reflected by Δ*I*(*t*)*/I*_0_ follows the same trend as the lattice expansion reflected by Δ*S*(*t*)*/S*_0_. Since the Δ*T*(*t*) of TiO_2_ (211) rises with a time constant of 2.62 ps, one might expect a comparable t_S1_ value for Δ*T*(*t*) of TiO_2_. However, t_S1_ exhibits a notably small value of 0.41 ps, which implies that the initial thermal expansion process of TiO_2_, which is faster than that of Au, was thwarted by the expanding Au. This assignment is also supported by the faster decay time constant of TiO_2_ (t_A1,TiO2_ = 2.62 ± 0.35 ps) than that of Au (t_A1,Au_ = 3.05 ± 0.13 ps). This ultrafast temperature rise and expansion of TiO_2_ invokes a strong resemblance to e–p coupling as opposed to p–p coupling. This distinction arises from the temporal aspect, given that p–p coupling typically transpires over an extended duration (∼10 ps), unlike the rapidity associated with e–p coupling (several ps). This observation leads to the intriguing proposition that the TiO_2_ expansion may serve as tangible structural evidence for the transfer of energized electrons from Au to TiO_2_. The consequential impact of this electron transfer is evidenced by the instantaneous elevation in the TiO_2_ lattice temperature immediately after time zero, which is consistent with the observations from previous research on charge transfer dynamics.^[^ ^55^ ^]^ This finding highlights the dominant role of electron transfer in inducing the thermal energy transfer and rapid expansion of TiO_2_, underlining the intricate e–p coupling dynamics at play in the system.

The contraction of the TiO_2_ lattice, in turn, seems to be driven by the expansion of the Au lattice, rather than transferred hot electrons inside the TiO_2_. Notably, the ratio between the vibrational amplitude of Au (220) (A_S,Au_) and TiO_2_ (211) (A_S,TiO2_) peaks (Table S2) is 2.26, similar to the inverse of the ratio between Young's modulus of Au (γ = 100 GPa)^[^ ^57^ ^]^ and TiO_2_ (γ = 228 GPa),^[^ ^58^ ^]^ which is 2.28. The observation of anticorrelated vibrational motion and the ratio between their vibrational amplitudes strongly suggests that the vibrational motion originates predominantly from one domain rather than simultaneously from both Au and TiO_2_. If the coherent vibrational motions were launched by the expansion of both domains, their vibrational motions would not be anticorrelated to each other, nor would they possess the same frequency. Therefore, the evidence points towards a more dominant contribution of a single domain in driving the observed coherent vibrational motion, providing valuable insights into the underlying dynamics of the system. In previous studies on coherent vibrations in metals, the origin of these coherent vibrations was attributed to the vibrational response of the metal domain. For instance, it was reported the activation of coherent breathing motion (10–20 ps) upon excitation of the plasmonic absorption band (∼800 nm) in plasmonic Au.^[^ ^45^, ^59^, ^60^ ^]^ Our measured vibrational period (T_S_) of 16.0 ± 0.3 ps not only shows excellent agreement with those breathing motions of plasmonic Au but also shows a remarkable similarity to the value expected for a 1D standing wave condition (∼10 ps).^[^ ^48^, ^49^, ^50^ ^]^ Based on this, it is possible to deduce a plausible scenario concerning the Au/TiO_2_ interface. Upon photoexcitation, hot electrons are generated and coupled with the Au lattice, leading to an expansion and coherent vibration of the Au lattice. Some of these hot electrons transfer to the TiO_2_ layer, inducing early lattice expansion in TiO_2_. Subsequently, the mechanical stress generated in Au propagates to TiO_2_, causing the contraction of the TiO_2_ lattice. The expansion of Au prevents the TiO_2_ lattice, which had already expanded due to the transferred electrons, from further expanding, resulting in its contraction. As a result, an anticorrelated coherent vibration arises between the TiO_2_ and Au lattices.

### 2.6 Interfacial Coupling

Previous works, employing spectroscopy and diffraction techniques, have revealed that the nonuniform distribution of hot electrons within metal thin films is responsible for the observed coherent vibrations. This nonuniform distribution results from irradiation with a pump laser at one end of the sample.^[^ ^48^, ^49^, ^61^ ^]^ Conversely, the absence of coherent motion upon excitation is intricately tied to the thickness of the layer. Previous studies showed that for this phenomenon to manifest, the sample's thickness must exceed both the electron mean free path and the skin depth. In the case of Au, the reported electron mean free path and skin depth are 37.7 nm and 17.7 nm, respectively.^[^ ^62^, ^63^ ^]^ Therefore, as a general rule, Au films with a thickness less than approximately 40 nm typically do not display coherent vibrational motion due to the even distribution of hot electrons, a consequence of the extended electron mean free path and skin depth. In this respect, the observed vibrational motion in the Au layer of the Au/TiO_2_ sample, which is only 15 nm thick, does not align with this explanation.

In Figure S4, we present the Δ*S*(*t*)*/S*_0_ and Δ*I*(*t*)*/I*_0_ for both the Au/TiO_2_ sample and a 15‐nm Au film (consisting solely of the Au layer). Notably, in Figure S4a, as discussed earlier, we observe a complete absence of vibrational motion in the 15‐nm Au film. This observation confirms a critical point: samples with thicknesses thinner than the critical threshold (∼40 nm) cannot bear the strong nonuniform distribution of hot electrons, resulting in relatively weak and fast dephasing coherent vibrations. Consequently, the weak nonuniformity of hot electrons within the 15‐nm Au sample launches relatively weak vibrational motion, leading to rapid dephasing of vibrational coherence in this sample.^[^ ^61^ ^]^ This absence of vibrational motion in the 15‐nm Au film aligns with explanations provided in prior studies.^[^ ^48^, ^49^, ^61^ ^]^ As a result, the vibrational behavior in the Au/TiO_2_ sample becomes even more intriguing and merits further investigation.

In this context, as the only distinguishing factor between the two samples is the presence of the TiO_2_ layer, it is plausible that the TiO_2_ layer played a crucial role in extending the lifetime of the coherent phonon, facilitating the manifestation of coherent vibrational motion in Au/TiO_2_. The presence of the neighboring TiO_2_ layer could potentially modify the mechanical motion of Au in two distinct manners: i) by affecting Au's mechanical behavior through the bulk properties of TiO_2_, and ii) by influencing mechanical motion at the interface through the modulation of interfacial properties between the two layers. For case i), prior studies using asynchronous optical sampling demonstrated that the coherent acoustic phonon lifetime of Au can be increased in a self‐assembled monolayer.^[^ ^64^ ^]^ This phenomenon is explained by the visco‐elastic model, where the coherent phonon lifetime can be modulated by the loss coefficient or elastic property of the material attached to Au.^[^ ^65^ ^]^ Interestingly, based on our calculation, we found that although the substrate materials, Si_3_N_4_ and TiO_2_ have similar mechanical properties (elasticity, loss coefficient, etc.) in the neutral state, the moduli of TiO_2_ decreases and TiO_2_ loses its stiffness as electrons are transferred to TiO_2_. This indicates the dephasing time of coherent vibrational motion, which is inversely proportional to the overall material stiffness, would be extended^[^ ^66^ ^]^ (Details on the calculation of bulk properties are discussed in the “**Computational analysis**” section). For case ii), regarding the Au/TiO_2_ interface, it is known that the adhesion between Au and TiO_2_ is weak due to the low polarizability of Au.^[^ ^16^ ^]^ Consequently, the interfacial coupling between Au and TiO_2_ in the unexcited, neutral, state is not particularly strong, making it hard to estimate the strengthening of vibrational coherence. On the other hand, we found that in the charged state of TiO_2_, which is induced by hot electron transfer from Au, interfacial p–p coupling could be enhanced by the activation of low‐frequency phonons in TiO_2_. In this regard, to investigate this interlayer charge transfer and phonon activation process, we conducted ab‐initio calculations to investigate the mechanical and thermal properties of TiO_2_, uncovering a noteworthy phenomenon: the electron transfer triggers the emergence of low‐frequency phonons in TiO_2_ greatly enhancing the overlap to the phonon modes of Au (Figure 5c). These findings strongly suggest that the coherent vibrational motion observed in Au/TiO_2_, which is shown in Figure 3a, is prompted by charge transfer at the interface. Specifically, the transfer of hot electrons to TiO_2_ significantly alters both i) the mechanical behavior of both Au and TiO_2_ by modifying TiO_2_’s bulk properties and ii) interfacial p–p coupling between Au and TiO_2_ by enhancing the overlap between vDOS of Au and TiO_2_ layers. This modulation activates phonon modes of both Au and TiO_2_ at the interface and impedes the rapid dephasing of vibrational motion by active mechanical interactions at the interface. Furthermore, hot electron transfer plays a pivotal role in shaping the behavior of the entire Au/TiO_2_ heterojunction system. A more comprehensive discussion of the theoretical calculation of these mechanisms will be provided in the “Computational analysis” section.

**Figure 4:**
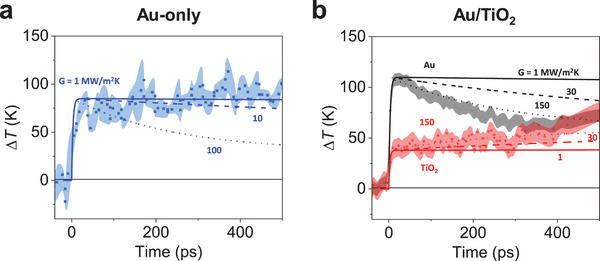
Temperature changes in Au‐only and Au/TiO_2_ samples and simulated temperature changes. To investigate the modulations in the interfacial thermal conductance, FEM simulations were performed. (a) Experimentally measured temperature changes of Au (220) peak of 15‐nm Au film (blue dot) and simulated temperature changes (blue lines) in the existence of SiN membrane with various thermal conductance (G) between Au and SiN are shown. (b) Time‐resolved temperature changes in Au (220) and TiO_2_ (211) peaks in Au/TiO_2_ sample (black dot and red dot, respectively) and simulated temperature changes Au (black lines) and TiO_2_ (red lines) in the existence of both SiN and TiO_2_ domain are shown. For these simulations, thermal conductance between Au and SiN was fixed to 1 MW/m^2^K and thermal conductance (G) between Au and TiO_2_ was varied. Data are presented as solid dots with the standard deviations as shaded areas. Experiments are conducted using an 800 nm pump pulse with 2.2 mJ/cm^2^ fluence. The simulation suggests that the interfacial thermal conductance governs the temperature dynamics of each layer. Thermal conductance decreased between Au and SiN, while it increased between Au and TiO_2_.

One of the crucial aspects is the degree of temperature change in each layer, which contains information on the number of electrons transferred to the TiO_2_ layer upon excitation. Since the thermal energy gained by TiO_2_ likely originates from transferred hot electrons, the thermal energy of the TiO_2_ layer reflects the fraction of transferred electrons among the total hot electrons resulting from photoexcitation. Notably, in Figure 3b, the temperature of TiO_2_ rises to half that of Au, showing the initial rise in the temperature of Au and TiO_2_ are 89 K and 45 K, respectively. And the ratio between the areas of Au and TiO_2_ domains turns out to be approximately 0.67 (see Figure S5). From these data, we can calculate the ratio of heat capacities per unit area of two layers (C_Au_/C_TiO2_ = 0.43) and the ratio of the total energies dissipated in two layers (Q_Au_/Q_TiO2_ = 0.84). As the transferred hot electrons release their energy in the conduction band of TiO_2_, situated 0.6 ∼ 0.7 eV above the Fermi level, while the remaining untransferred hot electrons dissipate their energy at the Fermi level, the thermal energy generated from hot electrons in each layer varies: approximately 1.54 eV for Au and 0.8 ∼ 0.9 eV for TiO_2_.^[^ ^67^ ^]^ Consequently, the hot electron injection yield from Au to TiO_2_ is calculated to be approximately 68% (under a laser fluence of 1.6 mJ/cm^2^), which is comparable to that of previous studies.^[^ ^68^ ^]^ In **Figure** **4**b, the temperature changes of Au and TiO_2_ of Au/TiO_2_ under 2.2 mJ/cm^2^ laser fluence are depicted. The electron injection yield at this fluence stands at 63%, exhibiting a slight decrease compared to the 1.6 mJ/cm^2^ case. In Figure S6, the electron injection yields corresponding to four different photon fluences are presented, revealing a consistent decrease in electron injection yield with increasing photon fluence.

Another intriguing aspect lies in the thermal conductance between layers. Figure 4 illustrates the temperature changes of each layer in both Au/TiO_2_ and Au‐only samples, alongside simulated temperature changes. Whereas the temperature of the Au‐only sample stays steady after rising, the temperature of Au and TiO_2_ of the Au/TiO_2_ sample decays exponentially and rises linearly, respectively. To evaluate how thermal energy propagates within the sample, Finite Element Method (FEM) simulations were performed on each sample system. Considering that SiN serves as the substrate, we also examined the impact of the SiN membrane on heat dissipation. For the simulation, we used reported values of heat capacity, density, and thermal conductivity for each domain (Au, TiO_2_, and SiN) and adjusted the thermal conductance between Au and TiO_2_ as well as between Au and SiN.

Figure 4a depicts the temperature change of the Au‐only sample. As this sample only involves contact between Au and SiN, the thermal energy of Au can solely dissipate into the SiN membrane. Simulation results reveal that the thermal conductance between Au and SiN exhibits a value of approximately 1 MW/m^2^K, significantly lower than the value reported in the literature (around 123 MW/m^2^K).^[^ ^69^ ^]^ We suspect that this large discrepancy between the measured value and the experimental value is due to the non‐equilibrated phonon energy distribution. While the reported thermal conductance between Au and SiN was measured in an equilibrium state, where they maintain a constant temperature and thermal energy flow, our system was pumped with an ultrafast pulse to excite the sample. In this ultrafast pumping regime, it takes a long time (∼100 ps) for the energies of phonons to achieve thermal equilibrium. Thus, because the phonon modes are not fully activated right after excitation, the delay in energy redistribution among phonons and subsequent energy transfer to TiO_2_ through interfacial p–p coupling would affect the thermal conductance on an ultrafast timescale.

**Figure 5:**
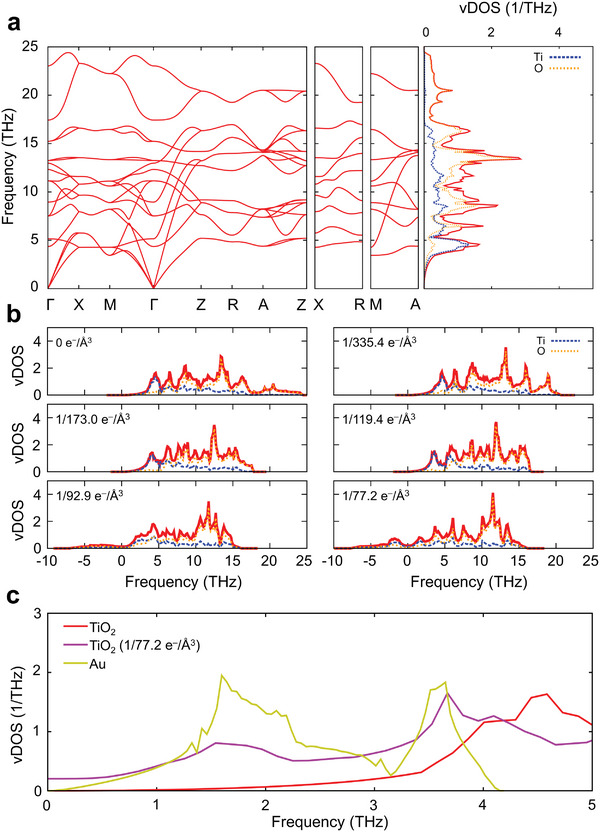
Phonon dispersion and vibrational density of states in rutile TiO_2_. (a) Calculated phonon dispersion and vibrational density of states (vDOS) for the optimized rutile TiO_2_ cell, highlighting atomic contributions from Ti^4+^ (Ti) and O^2−^ (O) ions, shown in blue and orange, respectively. The total vDOS is shown in red. (b) vDOS for neutral TiO_2_ (left upper panel) as well as for 5 different charged rutile TiO_2_ cells. The charged cells show a progression of extra electron concentrations per unit cell, as indicated in the left upper corner of each panel. (c) Comparison of low‐frequency vDOS between Au and neutral and charged TiO_2_ cells. The height of the Au vDOS has been scaled down by a factor of four to facilitate a clearer comparison with the TiO_2_’s vDOS.

As shown in Figure 4b, the Au/TiO_2_ sample shows completely different dynamics from the Au‐only sample. Despite similar thermal properties (e.g., heat capacity and thermal conductivity) of SiN and TiO_2_, the temperature of Au in Au/TiO_2_ experiences an exponential decrease after heating up unlike the constant temperature exhibited in the Au‐only sample. Meanwhile, TiO_2_ exhibits a continuous rise after the initial heat up, which is induced by e–p coupling with transferred electrons. Here, the FEM simulation results suggest that the thermal conductance of Au‐TiO_2_ has a higher value (approximately 150 MW/m^2^K) compared to that reported in the literature (72 MW/m^2^K).^[^ ^70^ ^]^ This significant improvement in thermal conductance can be attributed to the enhanced phonon overlap and strengthened p–p coupling between the phonon modes of both Au and TiO_2_, as demonstrated in our ab initio calculations (**Figure** **5**c). Moreover, the FEM simulation adeptly captures the contrasting temperature dynamics, the exponential decay in Au and the linear rise in TiO_2_, aligning closely with the experimental observations. The simulation results suggest that this different temperature dynamics primarily arises from two factors: i) the larger coverage and heat capacity of TiO_2_, and ii) the low thermal conductivity of TiO_2_. Due to TiO_2_’s greater coverage (as depicted in Figure S1) compared to Au, temperature fluctuations within TiO_2_ are smaller, even when the entirety of dissipated thermal energy is transferred to it. Additionally, the low thermal conductivity within TiO_2_ dictates the overall temperature dynamics of the TiO_2_ layer, resulting in a slower temperature response across the entire TiO_2_ domain, hereby causing the temperature increase of TiO_2_ to appear linear. To delve deeper into this phenomenon and its impact on the charge transfer from Au to TiO_2_, we employed DFT calculations, which will be thoroughly examined in the forthcoming “Computational Analysis” section.

### 2.7 Computational Analysis

To understand how hot electron injection influences the behaviors of TiO_2_, such as its vibrational, thermodynamic, electronic, mechanical, and structural properties, we utilized theoretical simulations based on density functional theory (DFT) calculations. First, we investigated how the number of injected hot electrons influences the structural and vibrational characteristics of TiO_2_. We performed structural optimization of the 6‐atom rutile (P4_2_/mnm) TiO_2_ primitive cell to refine both ionic positions and lattice parameters for both neutral and charged unit cells. In the latter case, we analyzed ten distinct cells with varying extra electron counts, ranging from 0.1 to 1 electron per unit cell. Figures S7 and S8 depict the influence of excess electrons on unit cell volume and ion spacing, respectively, indicating that hot electron injection causes isotropic expansion of the TiO_2_ unit cell and increases the ion spacing. This lattice expansion results in significant alterations to the vibrational properties of TiO_2_. The phonon dispersion curve and the vibrational density of states (vDOS) of the neutral TiO_2_ cell show frequencies ranging from approximately 0 THz to 25 THz (Figure 5a). The right panel in Figure 5a plots the projected vDOS, which reveals three distinct frequency ranges: low (0‐6 THz), intermediate (6‐17.5 THz), and high (17.5‐25 THz). In the low‐frequency range, Ti^4+^ species primarily influence soft modes; the intermediate regime has a mixed contribution from Ti^4+^ and O^2−^ species; while the high‐frequency range is predominantly defined by O^2−^ contribution.^[^ ^71^ ^]^

The vDOSs for the charged cells, depicted in Figure 5b and Figure S9, display distinct vibrational features. In fact, as the number of electrons within the rutile TiO_2_ systems increases, all frequency ranges undergo substantial variations. In the low‐frequency region (Figure S10), the vDOSs shift towards lower frequencies. Upon reaching an excess electron concentration of 1/104.1 e^−^/Å,^3^ soft modes bearing negative frequencies appear, indicating instability in the crystal lattice and its susceptibility to structural distortions. Additionally, this low‐frequency region reveals a significant contribution from O^2−^ ions accompanying the emergence of negative frequencies. The new low‐frequency soft modes align well with the frequency range of bulk Au soft modes (Figure 5c), and such an overlap likely enhances interlayer p–p coupling. This coupling is thereby accountable for the coherent vibrational motions observed post‐charge transfer and the enhanced thermal conductance between Au and TiO_2_.

We now turn our attention to the intermediate and high‐frequency ranges. The vDOSs in Figure 5b reveal that, as concentrations of excess charges increase, the intermediate and high‐frequency regions of the vDOSs gradually merge, consolidating into a narrower band in the range of 6–15 THz, beginning at an electron concentration of 1/92.9 e^−^/Å.^3^ Following the merging of these frequency regions, it becomes more challenging to distinguish between modes dominated by Ti^4+^ or O^2−^ characteristics.

By analyzing the electronic density of states shown in Figure S11 alongside the vDOSs in Figure 5, we clarify how the transfer of hot electrons leads to increased ion spacing and cell parameters in TiO_2_. Specifically, the electrons injected into the TiO_2_ conduction band are primarily found in Ti 3d orbital states. Figure S12 displays the computed band‐decomposed charge density for the states occupied by the extra electrons, highlighting that they are primarily localized on Ti states. Adding electrons to these Ti 3d states disturbs the charge balance. Due to the negative polarization of O^2−^ relative to Ti^4+^, this addition diminishes the strength of oxygen‐titanium ionic interactions, leading to subsequent bond elongation. This elongation shifts the fast stretching modes to lower frequencies, consequently narrowing the vDOS as previously discussed. The observed shift of phonon frequencies to lower values and the narrowing of the frequency spectrum in charged TiO_2_ are expected to significantly impact its mechanical and thermal properties. These changes suggest alterations in the internal lattice dynamics, crucial for the material's response to mechanical stress and heat conduction.

Figure S13 presents the computed heat capacity as a function of temperature for both the neutral and charged TiO_2_ systems, alongside experimental values for comparison.^[^ ^85^ ^]^ The introduction of extra electrons leads to an overall increase in the semiconductor's heat capacity. Specifically, the inset of Figure S13 shows that the molar heat capacity at room temperature rises from ～57 J/mol·K in the neutral state to ～67 J/mol·K in the charged state with 0.6 extra electrons per unit cell. This increase may also contribute to the slow thermal equilibration observed in Figure 4b. A possible explanation for the thermal behavior seen in Figure 4b is that the initial temperature rise in TiO_2_ results from the e–p coupling triggered by the injected electrons altering the crystal lattice. The subsequent gradual temperature rise could be influenced by the material's macroscopic properties, encompassing not just thermal conductivity but also heat capacity. Following charge injection, heat capacity undergoes an increase, impeding swift thermal equilibration and achieving an equilibrium state only after approximately 400 ps.

The injected charge significantly influences the mechanical properties of TiO_2_, leading to reductions in the bulk modulus, shear modulus, and Young's modulus, as reported in Table S3. This decrease suggests the material becomes less rigid and more prone to deformation, indicating that the negative charge reduces its resistance to external forces and increases its mechanical flexibility. This enhanced flexibility likely contributes to the mechanical stresses induced by charge transfer, eventually facilitating heat and energy transfer.

In conclusion, our computational study elucidates the profound interplay between charge transfer‐induced structural changes and the vibrational, mechanical, and thermal properties, underscoring the significant impact of electronic modifications on the functionality and dynamics of metal/semiconductor interfaces.

## 3 Conclusion

**Figure 6:**
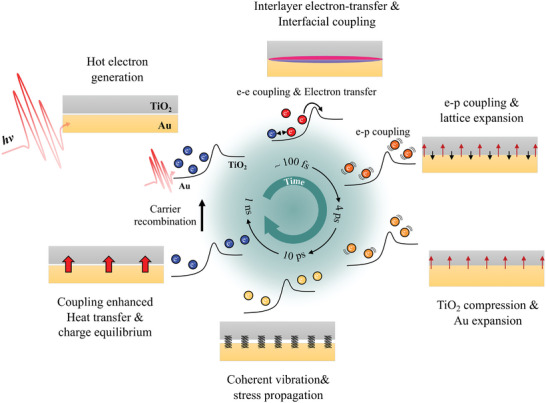
Schematic description of the charge and structural dynamics in the Au/TiO_2_ bilayer system. The Au and TiO_2_ layers are shown in yellow and gray, respectively. In the experiment, the Au/TiO_2_ bilayer was irradiated by an 800 nm pump pulse, which induced plasmonic absorption within Au, thereby generating hot electrons. A fraction of these hot electrons successfully surmounts the Schottky barrier and undergo interlayer electron transfer to TiO_2_, demonstrating rapid transfer dynamics on the order of ∼100 fs. The interlayer electron transfer makes TiO_2_ more deformable and initiates the coupling of phonon modes between Au and TiO_2_, leading to the interfacial coupling at the Au‐TiO_2_ interface. This coupling establishes a pathway for the propagation of mechanical stress and heat via the interface. Subsequently, the hot electrons in the Au and TiO_2_ start to dissipate their energy, heating up the lattice through electron–phonon coupling (∼1 ps). Meanwhile, both Au and TiO_2_ exhibit lattice expansion resulting from e–p coupling within their respective domains. This lattice expansion in Au leads to the generation of mechanical stresses, which are subsequently and efficiently transmitted to TiO_2_ through interfacial coupling, causing a contraction of the TiO_2_, which was softened by the transferred electrons (∼3 ps). These propagated stresses, in turn, initiate coherent vibrations between the two layers. Consequently, both the Au and TiO_2_ layers undergo coherent vibrational motion, exhibiting a 16 ps period, with their phases being anticorrelated (∼10 ps). Regarding the temperature of each layer, due to the difference in heat capacity of each layer, the temperatures of Au and TiO_2_ are not in equilibrium. However, owing to the enhanced thermal conductance at the Au‐TiO_2_ interface, the temperatures of Au and TiO_2_ reach equilibrium (∼1 ns). Finally, following a sufficiently extended duration (≫1 ns), the Au/TiO_2_ bilayer system returns to its ground state by dissipating heat into the environment and achieving charge equilibrium between the two domains.

The schematic in **Figure** **6** illustrates the photodynamics of the Au/TiO_2_ heterojunction system. In the Au/TiO_2_ system, hot electrons are generated through photoexcitation, as in the Au‐only sample, and are transferred to TiO_2_ via interlayer charge transfer. The remaining electrons in Au and those moving to TiO_2_ undergo lattice coupling in each domain, resulting in lattice expansion. Concurrently, the charge transfer modulates the bulk properties of TiO_2_ and induces interfacial p–p coupling at the Au‐TiO_2_ interface, enhancing the overlap of the phonon modes of Au and TiO_2_ in the low‐frequency region. This coupling reinforces the coherent vibrational motion at the interface, which is responsible for the observed coherent vibration. Moreover, this hot electron transfer‐induced interfacial coupling also plays a crucial role in thermal energy transfer by enhancing the thermal conductance at the Au/TiO_2_ interface. This behavior further corroborates the crucial role played by the interfacial coupling and coherent vibrational motion in the Au/TiO_2_ heterojunction system, enabling efficient thermal energy transfer and distinct thermal dynamics compared to the Au‐only sample. This distinct behavior further affirms the vital role played by the interfacial coupling and coherent vibrational motion in the Au/TiO_2_ heterojunction system. This unique combination enables efficient energy transfer.

By utilizing the UED technique, we have obtained evidence for the existence of a transient pathway for propagating stress and heat at metal‐semiconductor interfaces, facilitated by interlayer electron transfer. Interlayer charge transfer induces enhanced p–p coupling at the interface and makes TiO_2_ more receptive to coherent vibrations by modulating the mechanical properties of TiO_2_, highlighting the complex interplay between charge transfer and vibrational dynamics in this heterojunction system. This finding offers valuable insights that may significantly contribute to the engineering, design, and functionality of electronic devices.

## 4 Experimental Section

### Sample Preparation

Samples were prepared using the physical vapor deposition method. A SiN membrane (20 nm thick, Norcada) was used as the substrate. Using an e‐beam evaporator, Au and Ti layers were sequentially deposited on the SiN membrane. The deposited Ti layer was oxidized under 400 °C in the air for 2 hours, resulting in the formation of TiO_2_. Characterization of the resulting Au and TiO_2_ layers was performed using various techniques including UV‐visible absorption spectroscopy, X‐ray diffraction (XRD), scanning electron microscopy (Field Emission SEM, SU8230), and transmission electron microscopy (Titan Double Cs corrected TEM, Image / Probe Cs). To ensure precise control of the thickness during deposition, an in‐situ thickness monitor was employed. The deposition process continued until the Au layer reached a thickness of 15 nm and the Ti layer reached a thickness of 10 nm, as monitored by the in‐situ thickness monitor.

### Ultrafast Electron Diffraction

The experiment employed a custom‐built DC Ultrafast Electron Diffraction (UED) setup, wherein photoelectrons were accelerated through a DC accelerator and then compressed using RF fields within an RF cavity. It has been demonstrated that these emitted electron bunches can be compressed into femtosecond durations using an RF cavity buncher.^[^ ^72^, ^73^, ^74^ ^]^ Emitted electron bunches were accelerated with a 90 KV potential to reach the speed of ∼0.52 c with ∼400 fs time duration. **Figure** **S14** illustrates the schematic of the UED experimental setup. The Ti:Sapphire regenerative amplifier laser system (Spitfire of Spectra‐Physics) used in the UED setup generated sub‐35‐fs laser pulses within a wavelength tuning range of 770–830 nm, operating at a repetition rate of 1 kHz. The output pulse energy typically reached around 4 mJ. The laser pulses, centered at an 800‐nm wavelength, were split into pump and probe pulses using a beam splitter. The pump pulses, containing 50% of the original beam energy, were directed to the sample through a precision linear translation stage to excite the sample. The remaining 50% of the 800 nm pulses were sent to a third harmonic generator (THG). The tripled femtosecond pulses, with a photon energy of ∼4.65 eV (267 nm), were converted to femtosecond electron bunches via photoemission from the photo (copper) cathode and generated electron bunches are diffracted by the sample, capturing the temporal evolutions of structure. These transmission diffraction patterns are projected on a phosphor screen that was imaged onto an ICCD camera. The delay time between the optical pump beam and the electron probe beam was controlled by varying the relative optical path difference between the two beams.

### Data Processing

Time‐resolved two‐dimensional (2D) scattering images were acquired using an ICCD camera. The laser‐off images were repeatedly measured at a time delay of ‐30 ps for every scan. We performed two types of measurements with finer steps (200 fs and 1 ps) and coarser steps (6.6 ps). In the corresponding measurement, 400 and 500 images, respectively, were collected for every time delay on average. These 2D scattering images were then transformed into one‐dimensional (1D) azimuthally‐integrated scattering curves by computing the average intensity as a function of momentum transfer. The momentum transfer, denoted as *s*, is determined by the equation, *s* = (4π/λ)∙sin(2*θ*/2) = (4π/λ)∙sin[1/2∙tan^−1^(*l*/*d*)], where *λ* represents the de Broglie wavelength of the electron, 2*θ* denotes the scattering angle, *l* signifies the distance from the beam center to a specific pixel, and *d* represents the sample‐to‐detector distance. These collected azimuthally integrated scattering curves were then combined to generate the averaged 1D scattering curves. The resultant averaged 1D scattering curves were susceptible to contamination by systematic noise. To mitigate this issue, we employed the SANOD method on the 1D azimuthally integrated curves, resulting in corrected 1D curves that were subsequently employed for further data processing.^[^ ^75^, ^76^ ^]^

For data analysis, we utilized averaged 1D scattering curves to monitor both their peak intensities and peak positions. This process involved fitting the experimentally measured diffraction peaks with Gaussian functions.^[^ ^48^, ^49^, ^50^ ^]^ The optimization of peak parameters was achieved through the minimization of the χ^2^ value, which quantified the degree of discrepancy between experimental and theoretical Gaussian function, utilizing the MINUIT package developed at CERN.^[^ ^77^ ^]^ Error analysis was carried out using MINOS, an embedded algorithm within the MINUIT software.

### Ab Initio Calculations

We employed ab initio Density Functional Theory (DFT) calculations, using the Vienna ab initio simulation package (VASP) with projector augmented wave (PAW) pseudopotentials and a plane‐wave basis set,^[^ ^78^, ^79^, ^80^ ^]^ to explore the effects of injected hot electrons from Au to TiO_2_ on its structural and vibrational properties. All DFT calculations utilized a cutoff of 550 eV, high precision mode in VASP, and the PBE functional with a Hubbard U correction of 5 eV on Ti(d) states^[^ ^81^ ^]^ following Dudarev's approach.^[^ ^82^ ^]^ Additionally, Gaussian smearing with a width of 0.05 eV was applied to enhance density convergence.

We first performed structural optimization of a 6‐atom rutile (P4_2_/mnm) TiO_2_ primitive cell to refine ionic positions and lattice parameters for both neutral and various charged unit cells, spanning from 0.1 to 1 extra electron per unit cell. For all unit cells, the Brillouin zone (BZ) was sampled using a Γ‐centered 4 × 4 × 8 K‐points grid. Convergence was achieved when the energy difference between two consecutive electronic steps was below 10^−8^ eV and when the norms of all forces were less than 0.001 eV. First‐principles phonon calculations of rutile TiO_2_, including all charged cells, were conducted using the Density Functional Perturbation Theory (DFPT) method, as implemented in VASP. Vibrational properties were analyzed by expanding the optimized unit cells into 2 × 2 × 2 supercells and reducing the BZ sampling to a 2 × 2 × 4 K‐points grid. From DFTP calculations, we extracted the force constants and then computed the phonon modes (ω) using the Phonopy software package.^[^ ^83^ ^]^ These calculations employed a Monkhorst‐Pack grid of 30 × 30 × 30 q‐points for the phonon wave vectors. The resulting phonon modes were used to predict the phonon dispersion relations and the phonon density of states (vDOS) for the various TiO_2_ systems analyzed in this study. Additionally, Phonopy software was employed to calculate the heat capacity of TiO_2_ systems under the quasi‐harmonic approximation (QHA).^[^ ^83^ ^]^

We used the same computational setup to calculate the vDOS for the cubic Au system. The only difference from the calculations performed for TiO_2_ was that we began by optimizing the 4‐atom conventional cell, sampling the BZ using a Γ‐centered 8 × 8 × 8 K‐points grid. Subsequently, the phonon properties were evaluated using a 2 × 2 × 2 supercell, with the number of K‐points reduced accordingly.

Elastic constants (C_ij_) were determined using the strain‐stress relationship method with finite differences. This involved introducing six finite distortions to the crystal lattice to simulate various states of strain. For each state, the corresponding stress was computed to construct the elastic tensor. Subsequently, mechanical properties such as Bulk modulus (B), Shear modulus (G), Young's modulus (E), the B/G ratio, and Poisson's ratio (ν) were calculated using the Hill approach by using the VASPKIT software.^[^ ^84^ ^]^

## Conflict of Interest

The authors declare no conflict of interest.

## Author Contributions

H.I. directed the project. J.H., D.‐S.A. and H.I. conceived the idea, conceptualized and designed the experiment. J.H., D.K., D.‐S.A, K.Y.O. and S.P. designed and characterized the apparatus. J.H. and D.K conducted the UED experiment. J.H. prepared the sample. J.H. analyzed data and interpreted results. A.S. conducted theoretical simulations. J.H., A.S. and H.I. wrote the manuscript. All authors discussed the results.

## Supporting information

Supporting Information

## Data Availability

The data that support the findings of this study are available from the corresponding author upon reasonable request.
