# Supplementary material for: Ultrafast Interfacial Charge Transfer Initiates Mechanical Stress and Heat Transport at the Au‐TiO2 Interface
Source: Adv Sci (Weinh). 2024 Jul 8;11(34):2400919. doi: 10.1002/advs.202400919 (PMC11425853; doi:10.1002/advs.202400919)
Supplement: Supplementary file 1 — Supporting Information [file ADVS-11-2400919-s001.pdf]

ADVANCED SCIENCE
Open Access

Supporting Information

for *Adv. Sci.*, DOI 10.1002/advs.202400919

Ultrafast Interfacial Charge Transfer Initiates Mechanical Stress and Heat Transport at the Au-TiO_2 Interface

*Jun Heo, Alekos Segalina, Doyeong Kim, Doo-Sik Ahn, Key Young Oang, Sungjun Park, Hyungjun Kim and Hyotcherl Ihee**

Supporting Information

Ultrafast interfacial charge transfer initiates mechanical stress and heat transport at the Au-TiO_2 interface

Jun Heo, Alekos Segalina, Doyeong Kim, Doo-Sik Ahn, Key Young Oang, Sungjun Park, Hyungjun Kim, and Hyotcherl Ihee

The PDF file includes:

Figure S1 to S14

Table S1 to S3

Supporting Figures

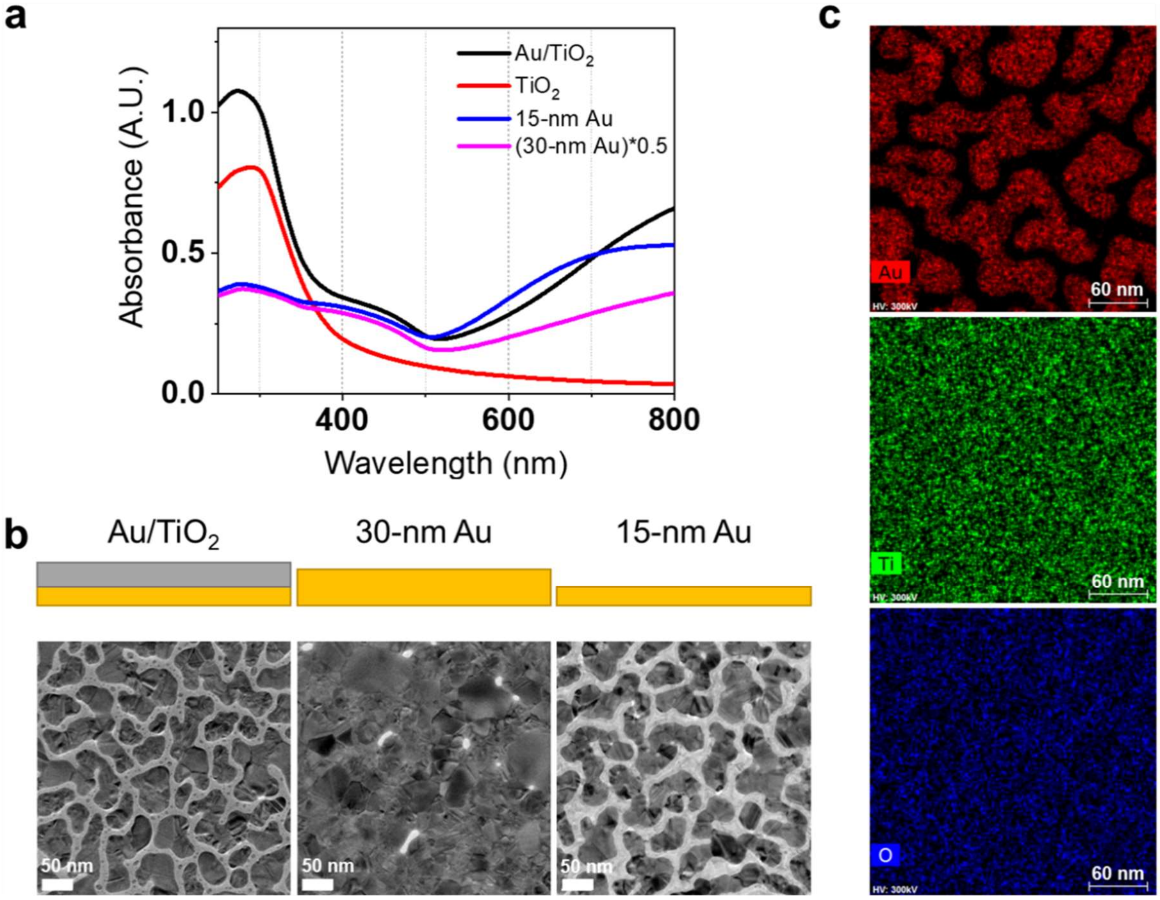

Figure S1. Characterization of the Au/TiO_2 bilayer sample. (a) UV-visible spectrum of Au/TiO_2 bilayer (black) compared with those of TiO_2 (red), 15-nm Au film (blue), and 30-nm Au film (magenta). The spectrum of 30-nm thick Au film is scaled down by a factor of two. (b) TEM image of Au/TiO_2 bilayer (left) compared with those of 30-nm Au film (center) and 15-nm Au film (right). At the top of each image, a schematic representation of its corresponding layer outline is presented. Au/TiO_2 bilayer and 15-nm Au film show similar Au morphology, unlike 30-nm Au film. The differences are consistent with their UV-visible spectra around 700 nm. (c) The elemental energy-dispersive X-ray spectroscopy (EDS) mapping images of Au (top, red), Ti (middle, green), and O (bottom, blue) for the Au/TiO_2 sample. The mapping images of Ti and O show that the TiO_2 layer is formed uniformly.

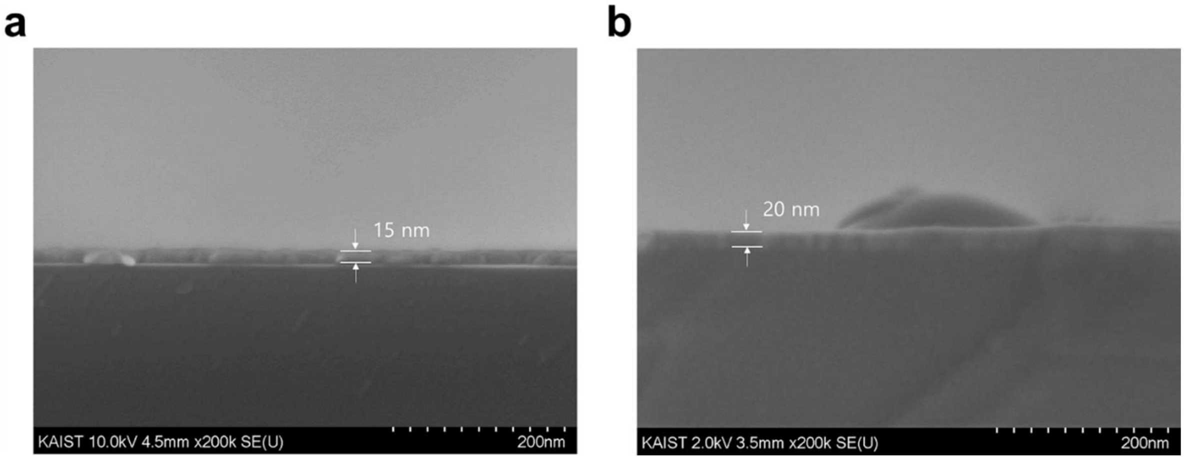

Figure S2. Cross-sectional SEM image of Au and TiO_2. (a) A cross-sectional view of a 15-nm-thick Au layer, which has a thickness of approximately 15 nm. (b) A cross-sectional view of a TiO_2 film with a thickness of 20 nm.

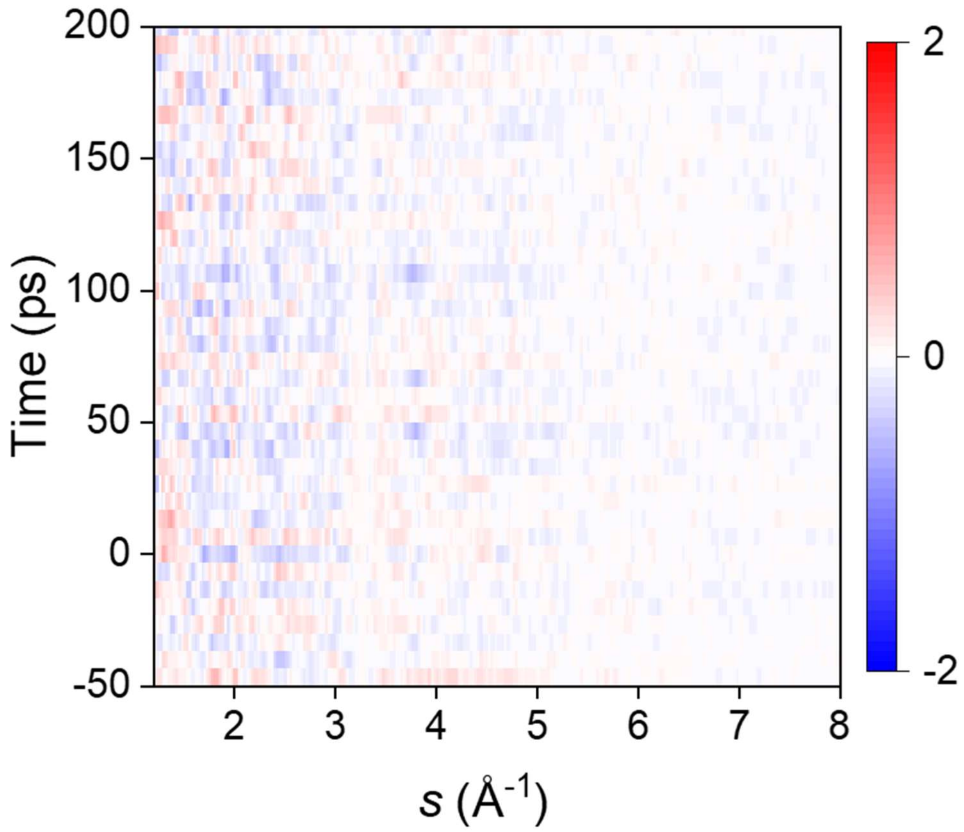

Figure S3. The experimental difference scattering curves of TiO_2 upon excitation with an 800 nm pump beam (0.6 mJ/cm^2). There are no apparent difference signals induced by the pump pulse. A color scale represents the intensity of the signal in an arbitrary unit.

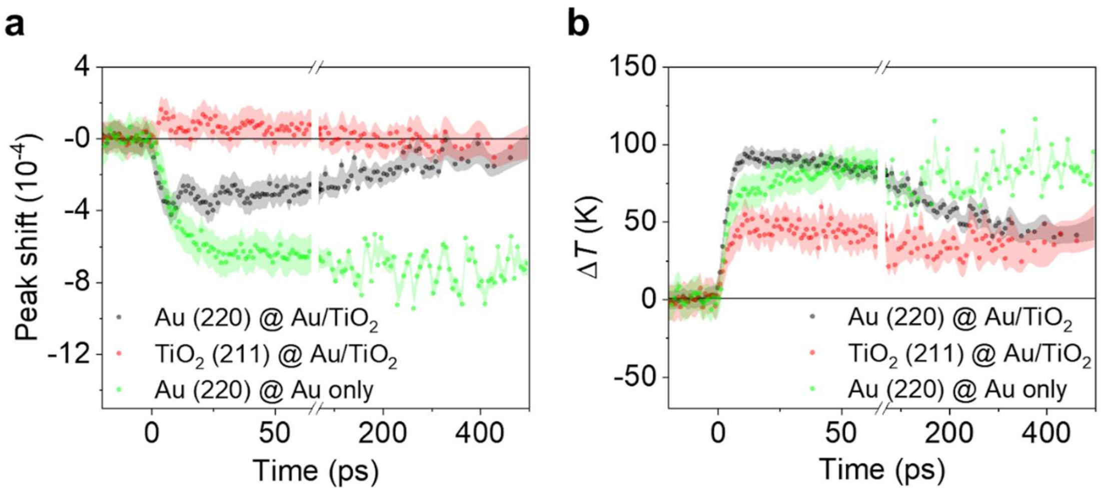

Figure S4. Long-term peak shift and temperature changes in Au-only and Au/TiO_2 samples upon photoexcitation. (a) Time-resolved peak shifts of Au (220) and TiO_2 (211) peaks of Au/TiO_2 sample (black dot and red dot, respectively) and Au (220) peak of Au-only (15-nm Au film, green dot) sample. (b) Time-resolved temperature changes in Au (220) and TiO_2 (211) peaks in the Au/TiO_2 sample (black dot and red dot, respectively) and Au (220) peak of the Au-only sample (green dot). Data are presented as solid dots with the standard deviations as shaded areas. Experiments are conducted using an 800 nm pump pulse with 1.6 mJ/cm^2 fluence.

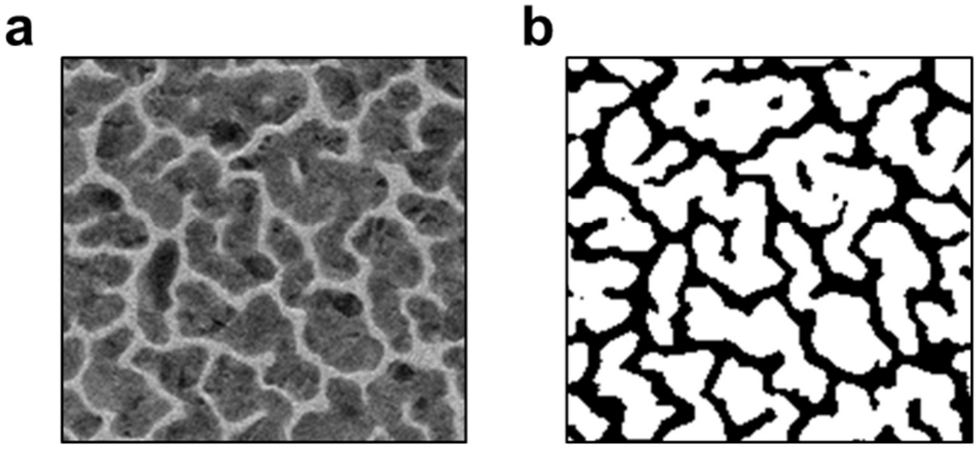

Figure S5. Image segmentation of a TEM image for the Au/TiO_2 sample. (a) TEM image of the Au/TiO_2 sample. (b) Segmentation of the Au domain is highlighted in white. The analysis reveals that Au covers 67% of the total sample area.

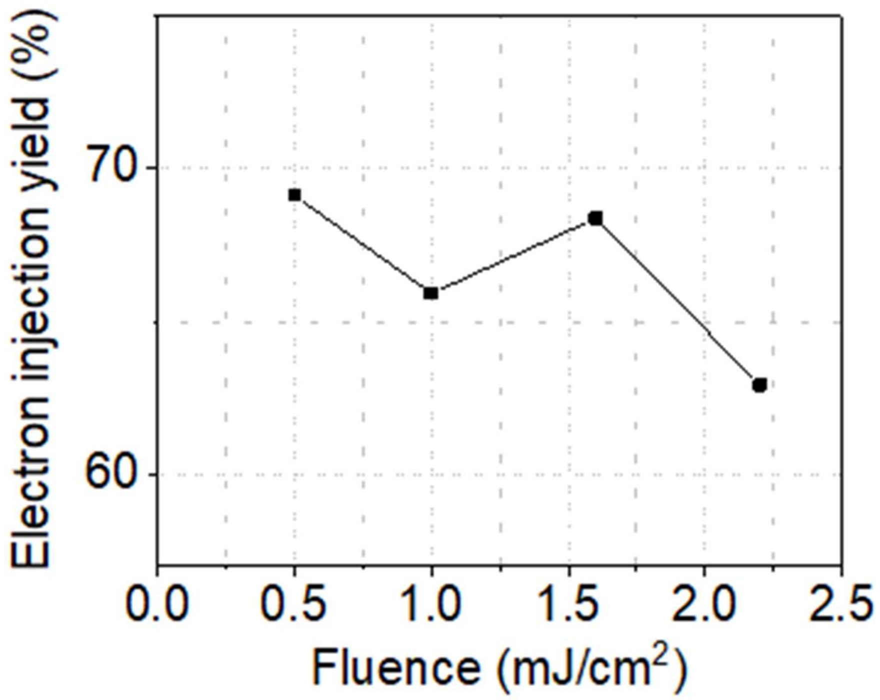

Figure S6. Fluence dependence of the electron injection yield from Au to TiO_2. Electron injection rates were calculated from the peak intensity changes shown in Figures 2a and 2c.

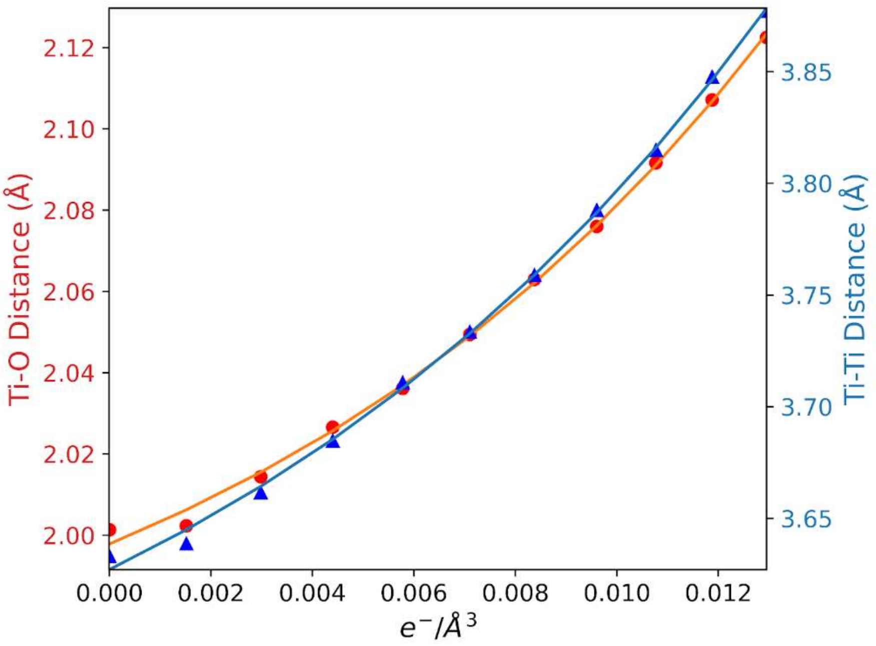

Figure S7. Relationship between the number of excess electrons within the TiO_2 unit cell and the averaged atomic distances. The averaged distance between Ti and O atoms (red circle) and Ti and Ti atoms (blue triangle) across various charge concentrations are plotted. Each distance alteration was fitted with an exponential function, with the resulting functions displayed as solid orange and blue lines for Ti–O and Ti–Ti distances, respectively. To accurately discern each distance, the cell parameters were fully optimized under each excess electron concentration.

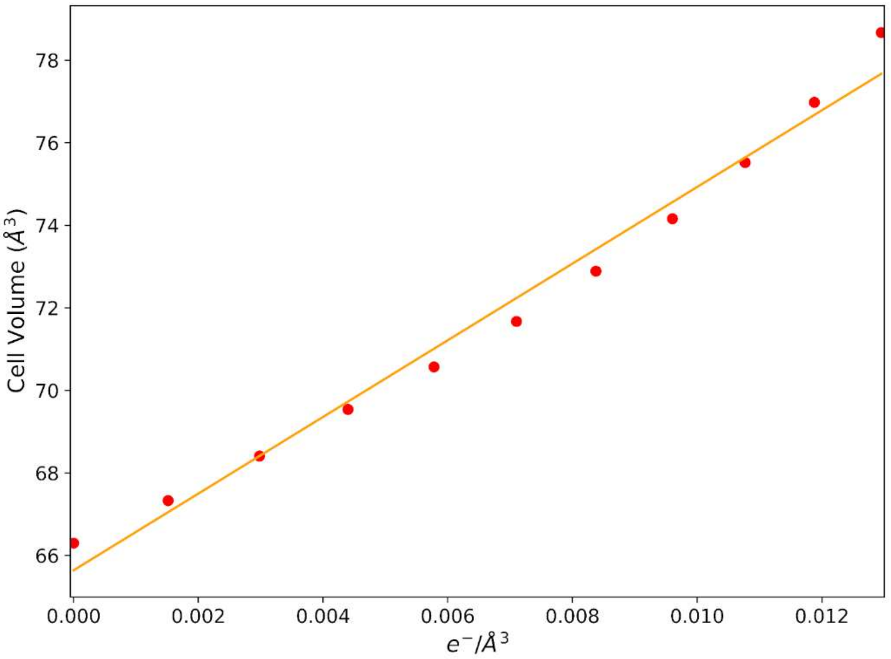

Figure S8. Relationship between the number of excess electrons within the TiO_2 unit cell and the volume of the unit cell. The volumetric changes in TiO_2 unit cells are plotted as a function of changes in the number of excess electrons (red circles). The volume change exhibits a linear dependency on changes in charge concentration, with the fitting function depicted as an orange solid line, obtained from fully optimized cell parameters.

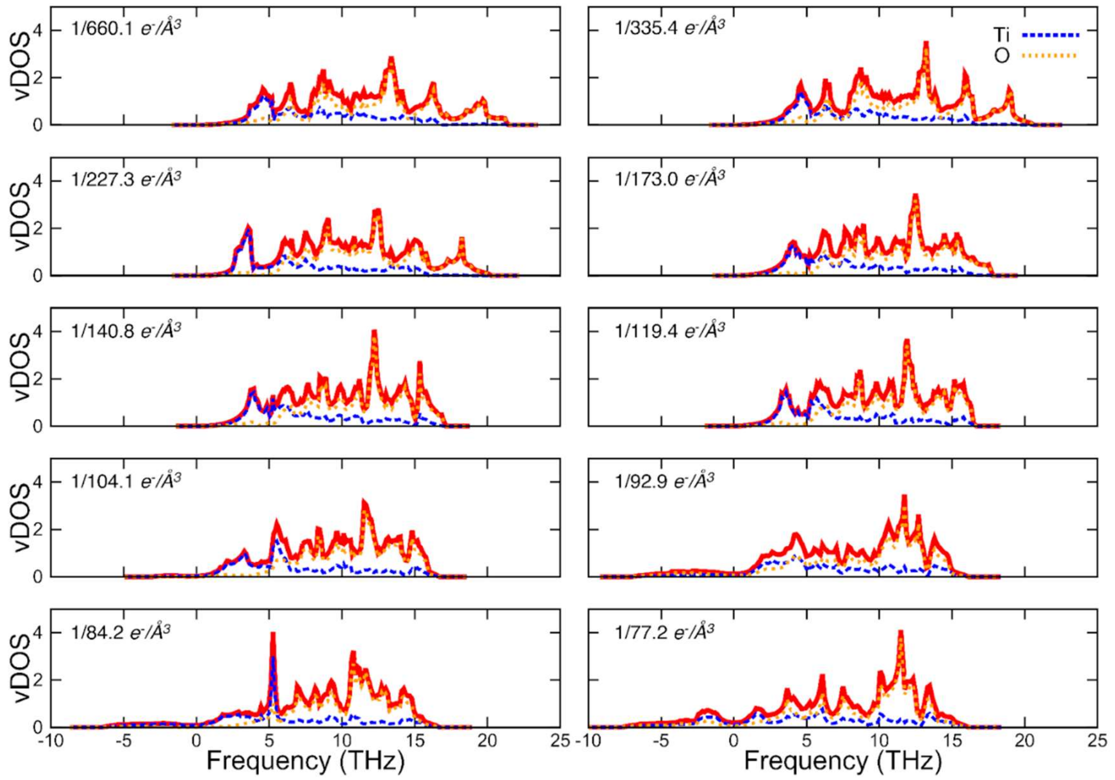

Figure S9. vDOSs for rutile TiO_2 depicting different concentrations of extra negative charge, ranging from 0.1 to 1 extra electron per unit cell, organized from the top left to the bottom right panel. Each panel highlights the specific concentration in the top left corner.

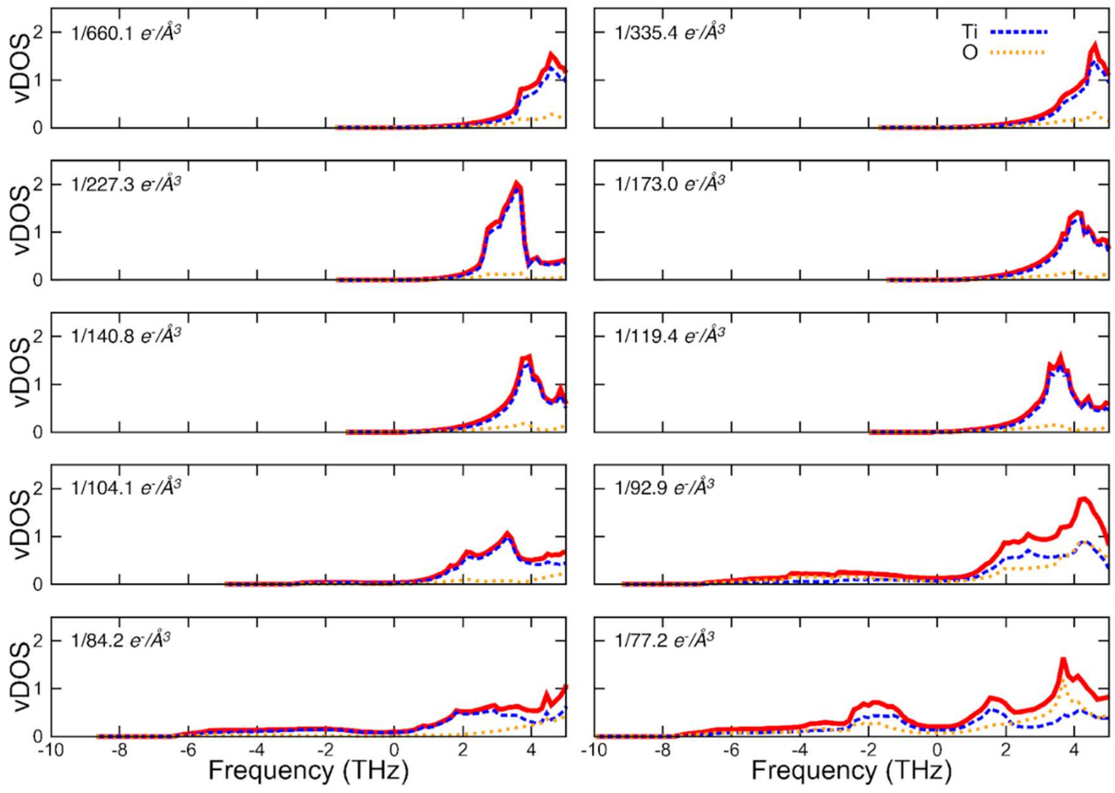

Figure S10. vDOS in the low-frequency region for rutile TiO_2 depicting different concentrations of extra negative charge, ranging from 0.1 to 1 extra electron per unit cell, organized from the top left to the bottom right panel. Each panel highlights the specific concentration in the top left corner.

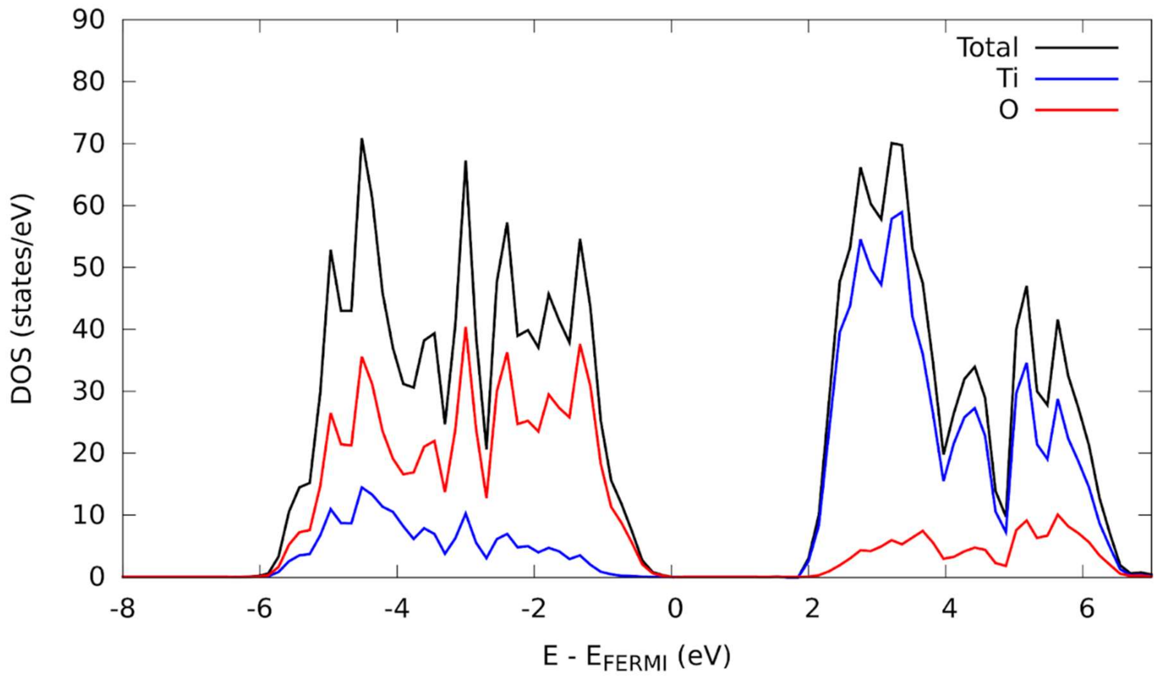

Figure S11. Electronic Density of States (DOS) and Projected Density of States (PDOS) for rutile TiO_2. The total DOS is depicted in black, while the PDOS for Ti states and O states are shown in blue and red, respectively.

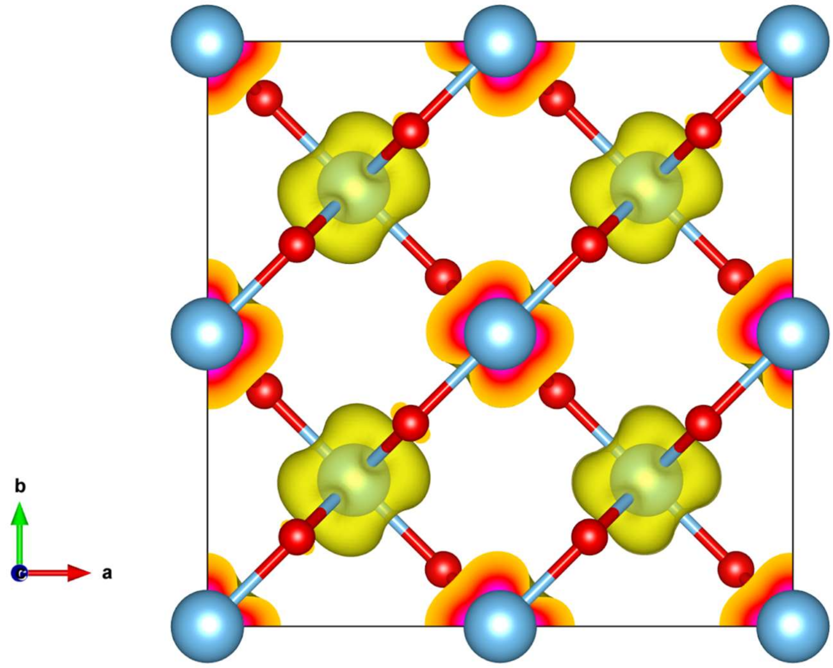

Figure S12. Band-decomposed charge density for the rutile TiO_2 system, illustrating the partial charge density within bands populated by excess charges. The image represents the charge density at an electron concentration of 1/335.4 $e^-/Å^3$, which is representative of the densities observed across all charged systems analyzed in this study.

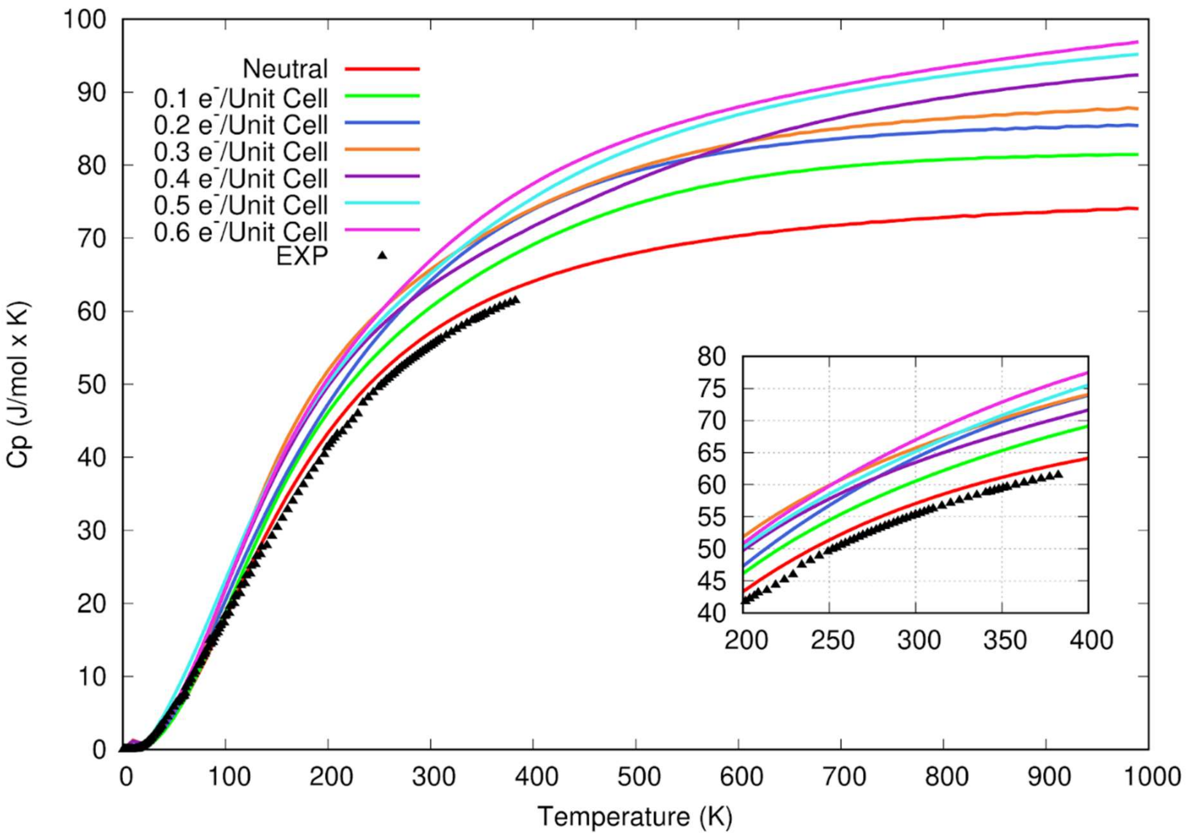

Figure S13. Calculated temperature-dependent molar heat capacity at constant pressure (Cp) of the rutile phase of TiO_2 for both the neutral system and the system with increasing extra electrons. The reported values are compared with experimental molar heat capacities at constant pressure, as adapted from reference[85].

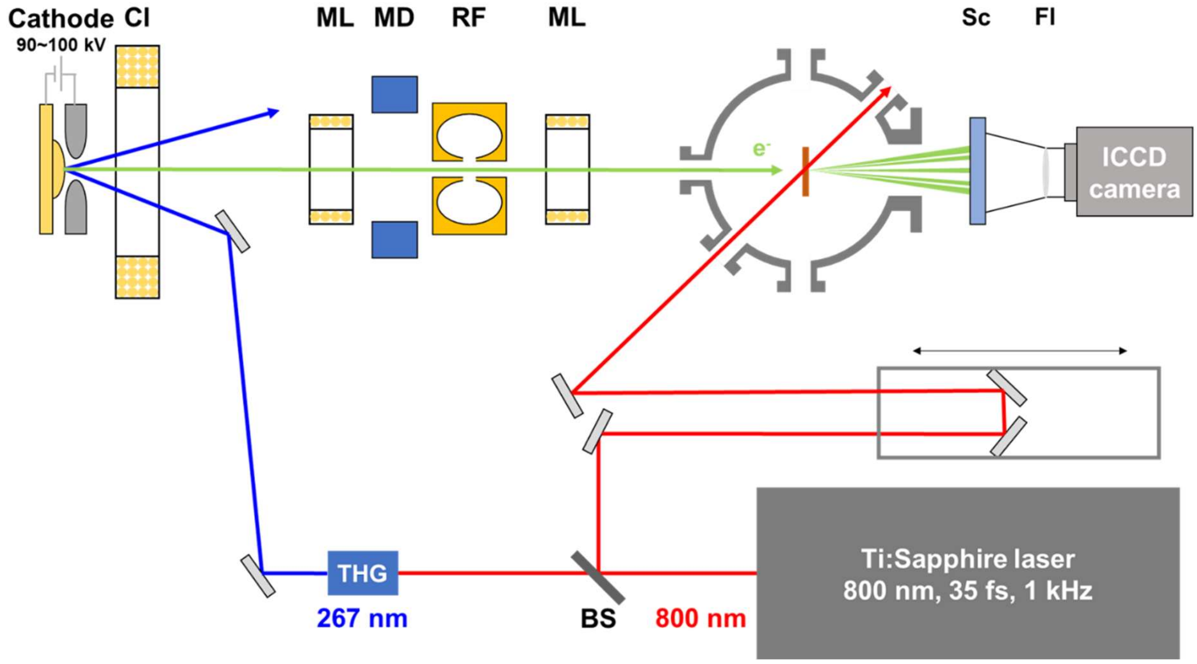

Figure S14. A schematic diagram of the UED experimental setup. Briefly, a Ti:Sapphire laser emitted an 800 nm beam (red) that was divided into two parts: one was used as a pump pulse, while the other was used to generate an electron pulse. To generate the electron pulse, the latter was subjected to third-harmonic generation to triple its frequency, and the resulting beam (blue) was then directed to a copper cathode. This process generated an electron bunch (green), which was manipulated by the downstream devices and used as the probe pulse. The abbreviations are as follows: BS = beam splitter, Cl = collimator, ML = magnetic lens, MD = magnetic deflector, RF = RF cavity, Sc = Scintillator, Fl = focusing lens, and THG = Third-harmonic generation.

Supporting Tables

Table S1. Fitted kinetic parameters of experimentally acquired peak shift and intensity change of Au (220) and the peak shift of TiO_2 (211) using exponential functions.

Fluence (mJ/cm^2)	Au peak shift (ps)	TiO_2 peak shift (ps)	Au peak amplitude change (ps)
0.6	249 ± 23	210 ± 92	118 ± 25
1.0	365 ± 29	127 ± 43	159 ± 43
1.6	433 ± 38	198 ± 71	201 ± 40
2.2	429 ± 25	219 ± 85	149 ± 17

Table S2. Fitted parameters of peak shift ($\Delta S(t)/S_0$) and temperature change ($\Delta T(t)$) of Au (220) and TiO_2 (211) peaks.

i) Peak position shift

<table>
<tr><th></th><th>t_{S1} (ps)</th><th>t_{S2} (ps)</th><th>t_{S3} (ps, fixed)</th><th>T_S (ps)</th><th>t_d (ps)</th><th>A_s (a.u.)</th></tr>
<tr><td>Au (220)</td><td>-</td><td rowspan="2">4.45 ± 0.79</td><td>433</td><td rowspan="2">16.0 ± 0.3</td><td rowspan="2">22.3 ± 4.0</td><td>$1.2\text{x } 10^{-4} \pm 1.4 \times 10^{-5}$</td></tr>
<tr><td>TiO_2 (211)</td><td>0.41 ± 0.34</td><td>198</td><td>$5.3 \text{ x } 10^{-5} \pm 6.9 \times 10^{-6}$</td></tr>
</table>

ii) Temperature change

	t_{A1} (ps)	t_{A2} (ps, fix)
Au (220)	3.05 ± 0.13	201
TiO_2 (211)	2.62 ± 0.35	-

Table S3. Computed mechanical properties of TiO_2 considering varying concentrations of excess electrons. The table presents Bulk modulus (B), Shear modulus (G), Young's modulus (E), the B/G ratio, and Poisson's ratio (*ν)* for each specified concentration, computed using the Hill approach.

Extra e^- conc. (e^-/$Å^3$)	B	G	E	B/G	**ν**
0	210	111.4	284.1	1.886	0.275
1/660	197.1	109.8	277.8	1.801	0.265
1/335.4	185	107.6	270.5	1.720	0.256
1/227.3	172.7	105	261.9	1.645	0.247
1/173	164.2	102	253.5	1.610	0.243
1/140.8	154.3	98.9	244.5	1.560	0.236
1/119.4	142.2	95.5	234.2	1.488	0.226
1/104.1	129.9	91.8	223.1	1.414	0.214
1/92.9	116.6	87.7	210.3	1.330	0.199
1/84.2	102.5	83	196.1	1.235	0.18
1/77.2	86.0	77.2	178.3	1.113	0.154
